# MazF6 toxin of *Mycobacterium tuberculosis* demonstrates antitoxin specificity and is coupled to regulation of cell growth by a Soj-like protein

**DOI:** 10.1186/1471-2180-13-240

**Published:** 2013-10-31

**Authors:** Melissa V Ramirez, Clinton C Dawson, Rebecca Crew, Kathleen England, Richard A Slayden

**Affiliations:** 1 Mycobacteria Research Laboratories, Department of Microbiology, Immunology, and Pathology, Colorado State University, Fort Collins, CO 80523, USA; 2 Current address: Department of Infectious Diseases, Stanford University School of Medicine, Stanford, CA 94305, USA; 3 Current address: Bacterial Diseases Branch, Division of Vector-Borne Diseases, National Center for Emerging and Zoonotic Infectious Diseases, Centers for Disease Control and Prevention, Fort Collins, CO USA

**Keywords:** *Mycobacterium tuberculosis*, Cell cycle, MazF, Toxin:antitoxin, Soj_Mtb_

## Abstract

**Background:**

Molecular programs employed by *Mycobacterium tuberculosis* (*Mtb*) for the establishment of non-replicating persistence (NRP) are poorly understood. In order to investigate mechanisms regulating entry into NRP, we asked how cell cycle regulation is linked to downstream adaptations that ultimately result in NRP. Based on previous reports and our recent studies, we reason that, in order to establish NRP, cells are halted in the cell cycle at the point of septum formation by coupled regulatory mechanisms.

**Results:**

Using bioinformatic consensus modeling, we identified an alternative cell cycle regulatory element, Soj_Mtb_ encoded by *rv1708*. Soj_Mtb_ coordinates a regulatory mechanism involving cell cycle control at the point of septum formation and elicits the induction of the MazF6 toxin. MazF6 functions as an mRNA interferase leading to bacteriostasis that can be prevented by interaction with its cognate antitoxin, MazE6. Further, MazEF6 acts independently of other Maz family toxin:antitoxin pairs. Notably, *soj*_*Mtb*_ and *mazEF6* transcripts where identified at 20, 40 and 100 days post-infection in increasing abundance indicating a role in adaption during chronic infection.

**Conclusions:**

Here we present the first evidence of a coupled regulatory system in which cell cycle regulation *via* Soj_Mtb_ is linked to downstream adaptations that are facilitated through the activity of the MazEF6 TA pair.

## Background

A hallmark of *Mycobacterium tuberculosis* (*Mtb*) is its ability to establish and maintain a latent infection capable of resuming growth after long periods of time during which no clinical symptoms of disease are evident. This substantial reservoir of latent infections with the potential for relapse to active disease and transmission presents a significant obstacle for successful disease management. Compounding this issue is the limited number of treatment options for chronic tuberculosis infections. Accordingly, there is a considerable need for new anti-tuberculosis therapeutics capable of treating both chronic and acute infections. However, before new drugs with novel modes of action can be developed more information about the coordination of adaptive metabolism associated with latency needs to be obtained.

The transition from rapid growth to the non-replicating persistent state (NRP) that is characteristic of chronic tuberculosis infection involves the downshift of multiple biological processes and a remodeled and reduced metabolism [1]. Previous studies utilizing *in vitro* models of *Mtb* NRP indicate the transition into this state occurs at a specific point during the bacterial cell cycle [1-3]. While these experimental observations highlight the required coordination of cell cycle progression with cessation of growth and metabolic shutdown, the precise regulatory mechanisms coupling cell cycle regulation with downstream global metabolic remodeling remain undefined.

Regulation of the cell cycle at the point of septum formation is a key checkpoint in coordinating bacterial stress responses and for transition into NRP [4-6]. The SOS response, which prevents cell cycle progression in response to DNA damage and other stresses, is a classic example of cell division regulation under conditional stress [7-16]. Although proteins that regulate septum formation have been widely characterized in other bacterial species, proteins that carry out this function have not all been identified or thoroughly characterized in *Mtb*[17]. A previous study from our laboratory identified a septum site determining protein, Ssd, and showed that it is capable of regulating septum formation and inducing alternative metabolic pathways, demonstrating a relationship between the regulation of cell cycle progression and the induction of molecular programs associated with NRP [6].

There is growing evidence that genome encoded toxin:antitoxin (TA) loci have important roles in *Mtb* biology and they are increasingly being associated with stress responses. TA loci encode an unstable antitoxin immediately upstream of a toxin and these proteins form a complex with one another under optimal growth conditions. This interaction prevents the toxin from exerting its effects on the specific cellular process it targets [18,19]. Several environmental stresses are known to result in the selective degradation of the antitoxin and accumulation of free toxin [20-23]. Of the 88 putative TA systems encoded in the *Mtb* genome, many have been shown to be responsive to hypoxia, participate in growth transitions, contribute to virulence, and regulate growth in macrophages or in sputum [20,24-29]. Yet, their regulation, coordination and specific contributions to the many stressful states encountered by *Mtb* are not fully understood [21,30].

Because of the large number of genome encoded TA loci, and lack of information about their regulation and interaction with other members of TA loci, our objective was to identify a cell cycle regulatory component, that was part of an alternative response that included TA loci, and to asses the interaction of the TA loci components. In this study, we identify Soj_Mtb_ encoded by *rv1708* as a cell cycle regulatory protein that governs cell cycle progression at the point of septum formation and elicits a complex adaptive response that includes the MazEF6 TA loci of *Mtb*. The MazF6 toxin is capable of inducing bacteriostasis that can be inhibited by interaction with its cognate antitoxin, MazE6. The MazEF6 TA pair form a stable protein complex and MazF6 is not capable of interacting with non-cognate antitoxins. This is the first report of a coupled regulatory system consisting of a cell cycle regulator, Soj_Mtb_, and a TA locus, that are involved in a global adaptive response ultimately associated with the transition into the NRP state of *Mtb*.

## Results

### ORF *rv1708* encodes a Soj-like protein in Mtb

**Figure 1:**
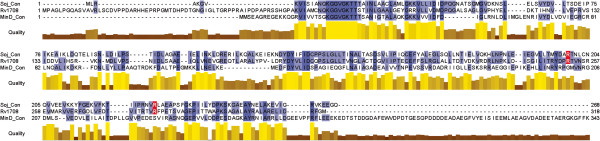
**Multialignment of Rv1708 with Soj and MinD consensus models.** Blue shading indicates conserved residues while red shading indicates residues involved in non-specific DNA binding by Soj proteins.

Previously, we used transcriptional mapping to assign *rv1708*, originally annotated as a putative initiation inhibition protein, as a septum formation regulatory protein [4]. To improve the functional assignment of *rv1708*, a bioinformatics approach using a consensus sequence derived from alignments of proteins with an annotated function of septum inhibition was employed. This substantiated the functional assignment of *rv1708* as encoding a septum regulatory protein because of shared similarity with the septum regulatory protein MinD. Septum inhibiting proteins, including MinD and Soj proteins, belong to the same family of P-Loop ATPases [31]. To narrow the functional assignment of Rv1708 we used the Phyre2 protein homology engine, which successfully revealed that Rv1708 shares greater sequence identity to Soj proteins (46% identity with 78% coverage; E value 2e-82) than to true MinD proteins (27% identity with 80% coverage; E value 6e-18). Alignments of the Rv1708 protein with Soj and MinD consensus models, Orthologous Matrix (OMA) groups 95838 and 78690 respectively, revealed that Rv1708 shares Soj-specific DNA-binding residues while lacking the hydrophobic C-terminal aliphatic helical extension that is involved in membrane association and is characteristic of MinD proteins [31,32] (Figure 1). Together, these analyses improve the functional annotation and indicate that *rv1708* encodes an alternative Soj protein, referred to here as Soj_Mtb_.

### Overexpression of *soj*_*Mtb*_ regulates cell cycle progression and acts prior to septum formation

**Figure 2:**
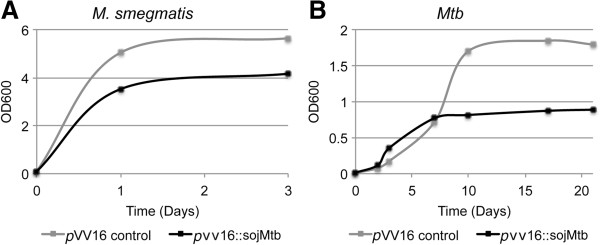
**Overexpression of*soj***_***Mtb***_**slows growth of mycobacteria.** Bacterial growth curves of **(A)***M. smegmatis* containing *p*VV16::*soj*_*Mtb*_ or *p*VV16 alone or **(B)***Mtb* containing *p*VV16::*soj*_*Mtb*_ or *p*VV16 alone. Data include technical replicates and are representative of three independent experiments.

**Figure 3:**
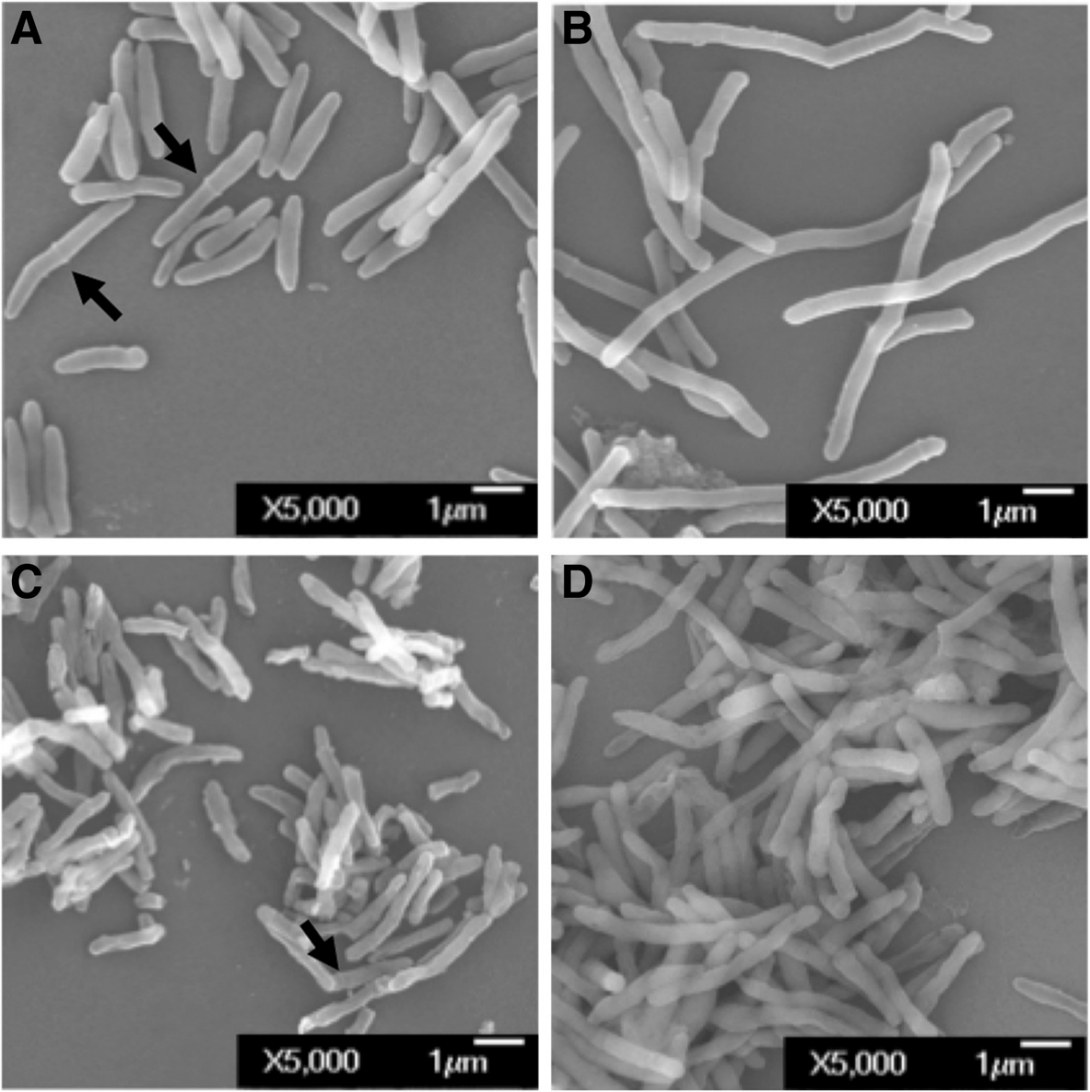
**Morphological characterization of mycobacteria overexpressing*soj***_***Mtb***_**. (A-B)***M. smegmatis* control and *soj*_*Mtb*_ merodiploid. **(C-D)***Mtb* control and *soj*_*Mtb*_ merodiploid.

To assess whether Soj_Mtb_ is involved in regulation of cell cycle progression, we performed gene dosage experiments. Overexpression of *soj*_*Mtb*_ caused a decrease in growth in *Mycobacterium smegmatis* and *Mtb* compared to the vector control strain (Figure 2). To visualize alterations in cellular morphology due to overexpression of *soj*_*Mtb*_, mycobacterial cells were imaged using electron microscopy (Figure 3). These studies revealed that overexpression of *soj*_*Mtb*_ in *M. smegmatis* and *Mtb* produced similar elongated cell phenotypes with a predominant ultra-structural morphology absent of concentric rings indicative of septa. This elongated cell phenotype is consistent with previous studies performed in *M. smegmatis*[33]. The absence of septa is characteristic of regulation of FtsZ polymerization prior to Z-ring formation [4,5,16,34,35]. Together, these data indicate that Soj_Mtb_ reduces the frequency of septum formation and governs cell cycle progression prior to septum formation.

### MazF6 toxin is elicited by Soj_Mtb_

**Figure 4:**
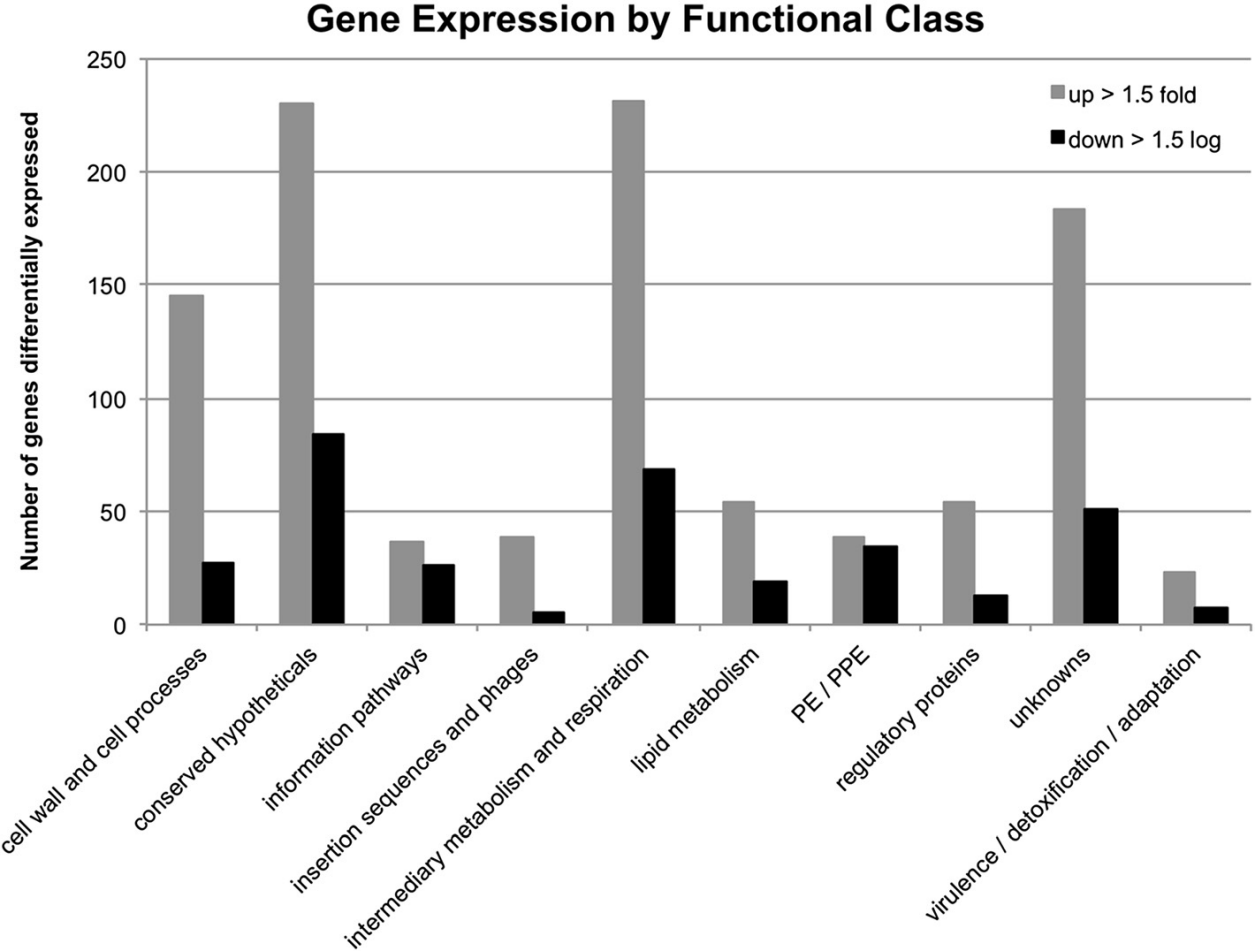
**The global transcriptional response of*Mtb*upon overexpression of*soj***_***Mtb***_**organized by functional class.** A total of 1,368 genes from all functional categories displaying a 1.5 fold or greater change in expression (p values < 0.05) in the *Mtb* merodiploid strain compared to the vector alone strain.

To assess the involvement of Soj_Mtb_ in eliciting alternative adaptive responses, the global transcriptional response of *Mtb* upon overexpression of *soj*_*Mtb*_ was investigated through whole genome DNA microarray analysis. A total of 1,368 genes from all functional categories displayed a 1.5 fold or greater change in expression (p values < 0.05) in the *Mtb* merodiploid strain compared to the vector alone control strain (Figure 4). The microarray data analysis revealed that Soj_Mtb_ is part of a large alternative stress response based on the induction of alternative sigma factors *sigH*, *sigG*, *sigL*, *sigM*, *sigI* in response to overexpression of *soj*_*Mtb*_. The pleotropic nature of this transcriptional response is likely the result of the induction of these alternative sigma factors, several of which have been shown to regulate stress response pathways [36]. Notably, *rv1991c* encoding the MazF6 toxin of the MazEF6 TA loci showed significant change in expression.

The MazF6 toxin is the most well characterized out of nine predicted *maz*-family TA loci encoded in the *Mtb* genome [20]. Molecular studies have demonstrated that the MazF6 is an endoribonuclease that cleaves ACA mRNA sequences, and that this activity is inhibited by interaction with the MazE6 antitoxin [37]. MazF6 is thought to induce rapid changes in the transcriptome due to its endoribonuclease activity, allowing coupling of cell cycle inhibition by Soj_Mtb_ and the transcriptional remodeling associated with the transition into an alternative state such as NRP.

We compared the *soj*_*Mtb*_ response with previously described programs associated with NRP in order to determine if there was overlap in the transcriptional responses. Overlap was determined by taking into account the number of genes in the given transcriptional response in relation to the number of genes in the genome (4,124 as indicated from the Broad Institute) to determine the number of genes that would be shared only by chance, as described previously [38]. There was no significant degree of overlap between the up-regulated genes of the *soj*_*Mtb*_ response and the *dos*-response or EHR (Table 1), and in fact, the *dos*-response was actively down-regulated (Table 2). However, there was significant overlap between the *soj*_*Mtb*_ response and the “non-culturable” response, with 14 up-regulated genes in common, the *mazEF6* transcript among them.

**Table 1:** **Comparisons of genes up-regulated upon overexpression of *soj***_***Mtb ***_**to previously described responses associated with NRP**

**Response associated with NRP**	**Soj**_**Mtb **_**response (923 up-regulated genes, over 2-log)**	
	**Overlapping genes**	**Overlapping by chance**
Dos^1^ (48 genes)	4	11
Enduring hypoxic response^2^ (230 genes)	44	52
Nonculturable^3^ (51 genes significantly up)	14	11

^1^[45], ^2^[39], ^3^[47].

**Table 2:** **Comparisons of genes down-regulated upon overexpression of** ***soj***_***Mtb ***_**to previously described responses associated with NRP**

**Response associated with NRP**	**Soj**_**Mtb **_**response (325 down-regulated genes, over 2-log)**	
	**Overlapping genes**	**Overlapping by chance**
Dos^1^ (48 genes)	21	4
Enduring hypoxic response^2^ (230 genes)	15	18
Nonculturable^3^ (51 genes significantly up)	4	4

^1^[45], ^2^[39], ^3^[47].

### MazE6 and MazF6 functionally and physically interact

**Figure 5:**
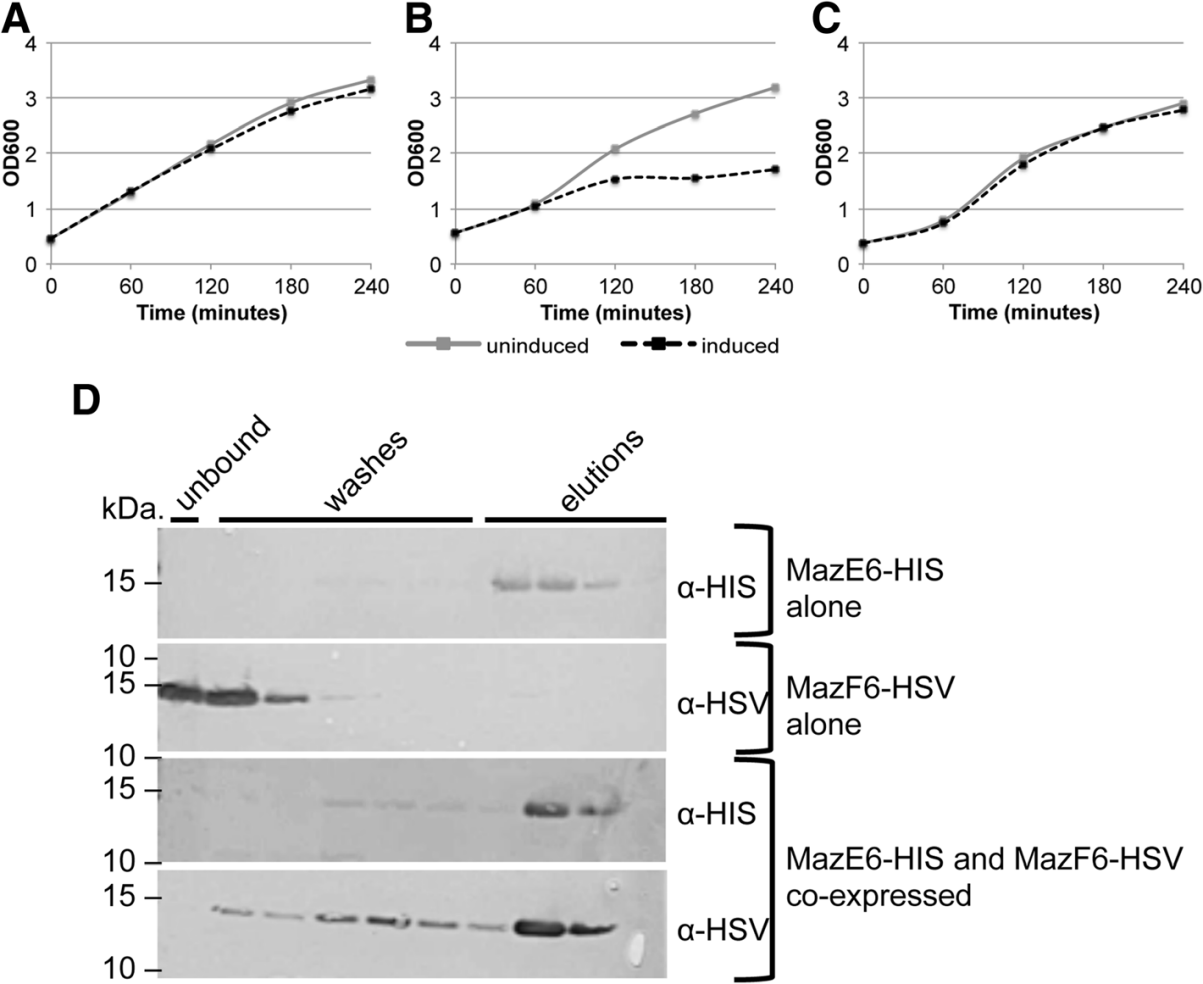
**MazE6-HIS antitoxin and MazF6-HSV toxin functionally and physically interact*in vivo.*A)** Growth of *E. coli* BL21 (DE3) pLysE containing MazE6-HIS alone, **B)** MazF6-HSV alone, or **C)** MazE6-HIS co-produced with MazF6-HSV. Growth arrest due to MazF6-HSV production is recued by co-production of MazE6-HIS antitoxin. **D)** MazE6-HIS is present in the elution fraction, while MazF6-HSV is present in the unbound flow and wash fraction when each is produced alone. When co-produced, MazF6-HSV elutes primarily with MazE6-HIS, indicating that they physically interact. Data are representative of multiple independent experiments.

To better understand the functional role of Maz-family TA pairs, we sought to characterize the functionality and interaction of MazE6 and MazF6. When expressed *in vivo,* the MazF6 toxin induces bacteriostasis, while expression of MazE6 causes no stalling of bacterial growth (Figures 4B and 5A, respectively). Importantly, this bacteriostatic phenotype can be rescued when the toxin and antitoxin are co-expressed thus demonstrating a functional interaction *in vivo* (Figure 5C). To show a physical interaction between these two recombinant proteins, co-purification was performed and protein identity was confirmed via Western blot and mass spectrometry. When both antitoxin and toxin are produced individually and subject to metal affinity chromatography, MazE6-HIS is present in the elution fraction, while MazF6-HSV is present in the unbound flow and wash fraction, indicating that it is not retained in the affinity column (Figure 5D). In contrast, when co-expressed, MazF6-HSV elutes primarily with MazE6-HIS indicating that they physically interact *in vivo* and that the bacteriostatic phenotype associated with overproduction of MazF6 can be prevented by its interaction with its cognate antitoxin, MazE6 (Figure 5). Western blot analysis was confirmed by in-gel trypsin digestion of the gel fragment corresponding to the recombinant MazEF6 complex and identification by LTQ mass spectrometry. MazEF6 were among the top hits returned when searched against the NCBInr database for eubacteria. MazE6 was identified with 70% coverage with 12 non-duplicate peptides, and MazF6 was identified with 95% coverage and 8 non-duplicate peptides.

**Figure 6:**
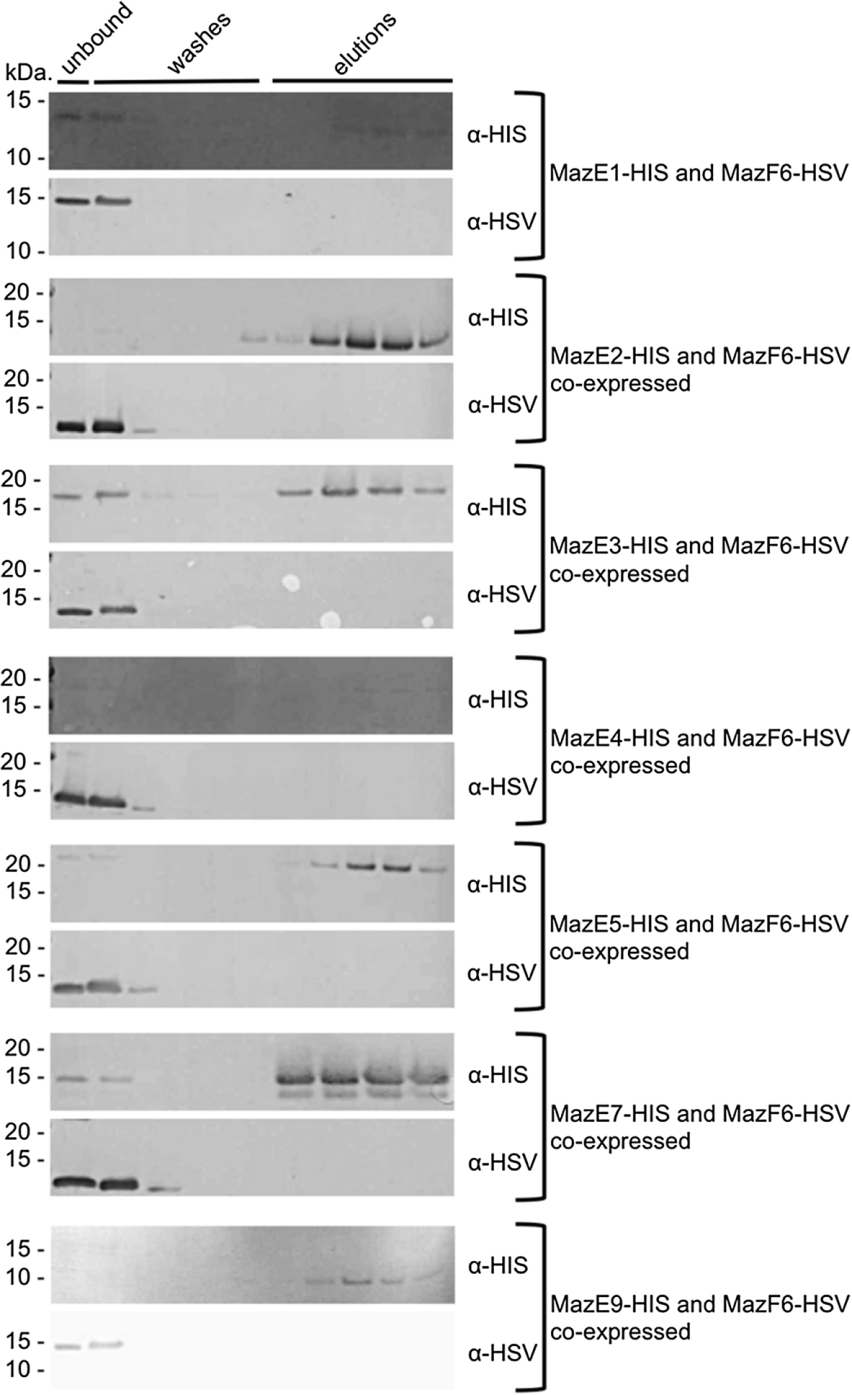
**MazF6-HSV toxin does not physically interact with non-cognate MazE-HIS antitoxins*in vivo.*** With all pairs tested, MazE-HIS antitoxins are present in the elution fractions, while MazF6-HSV is present in the unbound flow and wash fractions only, indicating that MazF6-HSV is only able to physically interact with its cognate antitoxin, MazE6. *From top to bottom, antitoxins tested are: MazE1 (Rv0456b), MazE2 (Rv0660c), MazE3 (Rv1103c), MazE4 (Rv1494), MazE5 (Rv1943c), MazE7 (Rv2063), MazE9 (Rv2801a).*

To determine if there is any degree of functional redundancy among the numerous MazEF loci in *Mtb*, we assessed the interactions between MazF6 and non-cognate MazE antitoxins. Functional redundancy was assessed by the ability of non-cognate MazE antitoxins to prevent bacteriostasis induced by MazF6. Growth arrest due to MazF6-HSV production was not rescued by co-production of non-cognate MazE-HIS antitoxins (Table 3). The MazF8 antitoxin encoded by *rv2274a* was not tested because it is absent in some clinical strains of *Mtb*[20,39]. Co-purification was also performed and with all pairs tested, MazF6-HSV was only able to physically interact with its cognate antitoxin, MazE6 (Figures 5D and 6). Our data confirm that the MazF6 toxin does not interact with non-cognate MazE antitoxins *in vivo* indicating that there may be little functional redundancy across MazEF pairs*.*

**Table 3:** **Growth kinetics in cultures co-expressing the *mazF6 *** **toxin with cognate and non-cognate antitoxins**

	**MazE**	**% Reduction in growth**
MazF6 expressed with non-cognate antitoxins	1	17
	2	30
	3	11
	4	17
	5	19
	7	33
	9	24
MazF6 alone	-	46
MazE6 alone	-	5
MazF6	6	4

Growth reduction associated with overexpression of the *mazF6* toxin can be rescued by co-expression of its cognate antitoxin. Full rescue of the growth defect is not observed when co-expressed with non-cognate antitoxins.

### *soj*_*Mtb*_ and *mazEF6* are expressed in bacteria in the lungs and spleen of *Mtb* infected mice

Adaptations resulting in a NRP state have been associated with the execution of complex and complementary molecular programs designed to allow the cell to survive the stresses experienced within the host [40]. These include programs to slow bacterial growth, induce alternative metabolic pathways, and to withstand acid, hypoxic, nutrient and additional stresses. TA loci have been associated with stress responses in bacteria and are becoming the focus of increasing efforts to elucidate their contributions to alternative states *in vivo*[18].

**Figure 7:**
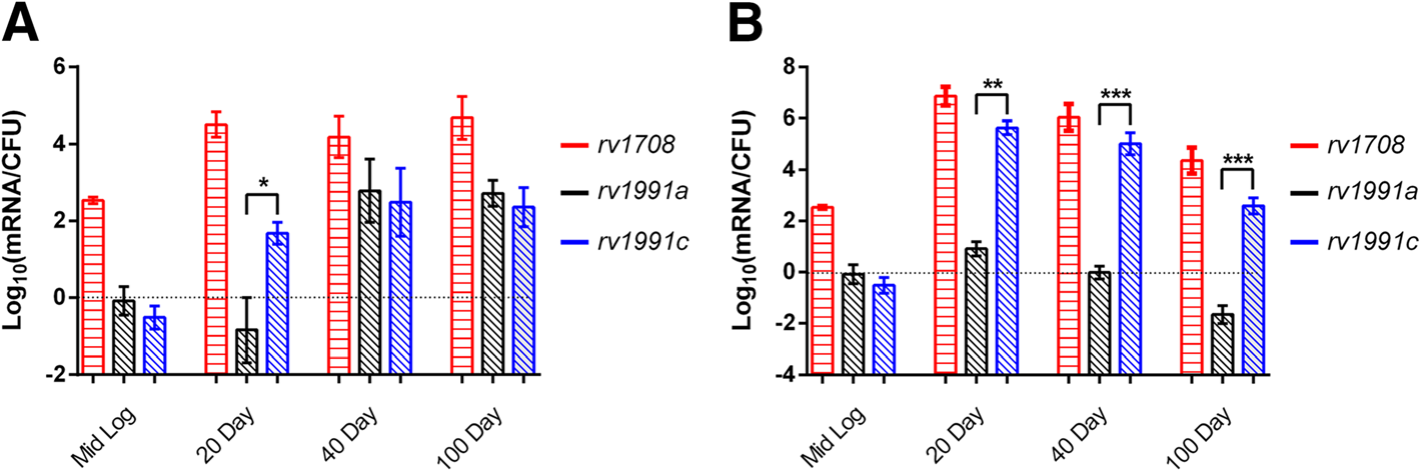
***soj***_***Mtb***_**and*mazEF6*are expressed during infection.** Quantative-PCR carried out on cDNA generated from RNA pools obtained from Mid-Log phase Mtb and mouse **(A)** lungs and **(B)** spleen at day 20, 40, and 100 post-infection. *soj*_*Mtb*_*(rv1708)* transcripts increased in abundance throughout the course of infection in comparison to Mid-Log phase Mtb. *mazEF6 (rv1991a, rv1991c)* transcripts were identified at day 20, 40 and 100 infection in the lungs and spleen.

In order to determine if Soj_Mtb_ and MazEF6 play a role during infection, we assessed their transcriptional activity during the infectious processes using the established mouse model of infection [41]. Quantitative-PCR was used to assess *soj*_*Mtb*_ and *mazEF6* abundance in cDNA obtained from *in vitro* Mid-Log phase cultures, and the lungs and spleen at 20, 40 and 100 days post-infection (Figure 7AB). The analyses revealed an overall difference in expression patterns and overall abundance of *soj*_*Mtb*_ and the *mazEF6* loci as compared to *in vitro* grown bacteria. *soj*_*Mtb*_ was abundant at day 20, 40, and 100. The transcriptional response in the lungs was characterized by a significant difference in the transcriptional abundance of *mazE6* and *mazF6* at day 20, while this difference in the abundance of *mazE6* and *mazF6* was not noted at days 40 and 100 (Figure 7A). Similarly to the transcriptional pattern observed at day 20 in the lungs, in the spleen *soj*_*Mtb*_ was abundant at day 20, 40 and 100, and there was a two log or greater difference in the abundance between *mazE6* and *mazF6* throughout the course of infection (Figure 7B). The observed transcriptional abundances observed between *mazE6* and *mazF6* is consistent with observations of TA loci in other systems [42]. Specifically, only the toxin component of several TA loci were identified in *Mtb* in a human macrophage model of late stage infection [43]. The transcriptional activity of *soj*_*Mtb*_, and *mazE6* and *mazF6* in bacteria obtained from infected tissues indicate that MazF6 is important throughout the course of infection, and is uniquely temporally functional in facilitating adaptations to the alternative environments encountered during infection.

## Discussion

The establishment of the NRP state requires the coordination of several important cellular processes. These include the halting of cell cycle progression at the point of septum formation, induction of alternative metabolic pathways, and coordination of multiple regulatory mechanisms, including TA loci, in modulating this adaptive response. Previous studies have observed that during macrophage infection, as well as under *in vitro* models of NRP, the bacteria are observed as multinucleoidal smooth filaments, indicating the transition into NRP occurs after chromosomal replication but prior to formation of the Z-ring septal precursor [1-3]. Although proteins that carry out this function in *Mtb* have not all been identified or characterized, we predicted that they must be present in the *Mtb* genome, as this is a critical checkpoint in the cell cycle. Homology searches for cell division regulators known to act by inhibiting septum formation identified *rv1708* (*soj*_*Mtb*_). Septum inhibiting proteins including MinD, ParA and Soj, show extensive similarity while participating in a variety of different cellular processes [31]. Homology models and local sequence alignments confirm that *rv1708* encodes a Soj-like, rather than MinD-like protein, as it contains the non-specific DNA-binding residues highly conserved in Soj proteins but lacking in MinD proteins [32]. While Soj and chromosomal ParA proteins are considered to be equivalent in respect to their roles in chromosome segregation, Soj proteins play an important role in the regulation of sporulation-specific gene expression and entry into stationary phase [44]. Previously, studies conducted in *M. smegmatis* reported that *rv1708* encodes a ParA homolog [33]. However, based on genomic context, we do not believe that the protein encoded by *rv1708* contributes significantly to chromosomal segregation as ParA.

Together, our bioinformatic analysis and morphological studies indicate a role in regulating cell division and demonstrate that Soj_Mtb_ performs a role in *Mtb* that is analogous to Soj proteins described in other bacteria. Soj_Mtb_ regulates the expression of a subset of genes that act to establish a state of low metabolic activity and reduced growth rate along with increasing adaptive functions required for entry into NRP. Transcriptional analyses of *Mtb* under a variety of conditions have uncovered several molecular programs that have been extensively linked to the NRP state of *Mtb* including the *dos* (*dormancy*) response, the enduring hypoxic response (EHR) and the “non-culturable” transitional state [45-48]. Our previous studies have demonstrated that septum site determining protein Ssd causes the induction of alternative metabolic pathways, including the *dos* regulon, further illustrating an intimate coordination of cell cycle progression with induction of molecular programs associated with NRP [6,45].

Upon overexpression of *soj*_*Mtb*_, alternative sigma factors *sigH*, *sigG*, *sigL*, *sigM*, *sigI*, and *sigB* are up-regulated. The pleotropic nature of this transcriptional response is likely the result of the induction of these alternative sigma factors, several of which have been shown to regulate stress response pathways [36]. In addition to the up-regulation of alternative sigma factors, the greatest increase in gene expression is seen for *rv1991c*, encoding the MazF6 toxin. A 2009 study characterized the transition to NRP by evaluating the “non-culturable” phenotype that has been associated with dormancy in *Mtb*[47]. Notably, *mazF6* was significantly up-regulated in this physiological model. In fact, there is significant overlap in the differential expression patterns between the “non-culturable” response and the response observed upon overexpression of *soj*_*Mtb*_. There was no significant degree of overlap between the up-regulated genes of the *soj*_*Mtb*_ response and the *dos* response or EHR, and in fact, the *dos* response was actively down-regulated. The *dos* response contributes to the preparation of *Mtb* for NRP, while the EHR contributes to the maintenance of NRP [46]. Although the *dos* response has been shown to aid in the adaptation to low oxygen state associated with NRP, mutants in the response have shown varying degrees of phenotypes and suggest that it does not play as prominent a role in NRP as once thought [38]. This further supports the role of the *soj*_*Mtb*_ response in preparing the bacterium for alternative conditions in a manner that is distinct from the preparation elicited by the *dos* response. Therefore, we posit that MazF6 functions to facilitate the transition into NRP. Likely, the endoribonuclease activity of MazF6 is utilized to erase the existing transcriptome to free cellular machinery to produce transcripts needed for successful entry into NRP.

Currently, there are nine predicted *maz*-family TA loci encoded in the *Mtb* genome [20]. Despite a number of reports, the information about the interaction of cognate and non-cognate Maz-family TA pairs is limited [49]. In this study we have demonstrated that the MazF6 toxin is capable of inducing bacteriostasis and that this state can be inhibited by interaction with its cognate antitoxin, MazE6. In addition, we have confirmed a physical interaction between these two proteins by purifying the proteins in complex from live cells. We have also shown that the interaction is specific and that MazF6 is not capable of interacting with non-cognate antitoxins. This suggests that although there are a high number of TA loci in *Mtb*, cross-talk *via* interaction is unlikely to occur, at least among this family of TA pairs. The identification of both *soj*_*Mtb*_ and *mazEF6* transcripts in bacteria isolated from the lungs and spleen of infected mice further supports the role of *soj*_*Mtb*_ and this toxin in facilitating the transition to alternative host environments through a coupled regulatory mechanism. The unique differential temporal profile of transcriptional abundance of the *mazEF6* polycistron throughout the host infection is concordant with previous observations. Post-transcriptional regulation of TA loci that are co-transcribed is a complex dynamic phenomenon that results in the accumulation of the toxin component of the bicistronic mRNA during times of environmental stress. A study of *Mtb* during human macrophage infection identified only the toxin component of several TA orthologs during late stage infection [43]. Similarly, a study in *E. coli* substantiated post-transcriptional modification of endogenous *mazEF* mRNA in the presence of RelE toxin resulting in the accumulation of *mazF* toxin mRNA [42]. Together with previous reports, our data indicate that regulation of TA loci is not strictly occurring at the post-translational level but also at the post-transcriptional level.

## Conclusions

All environmental conditions or cellular processes that induce the accumulation of the toxin component of TA loci or the specific phenotypes that results have not been fully elucidated. The identification of Soj_Mtb_, whose overproduction elicits the induction of this particular toxin loci, links the halting of cell division prior to the point of septum formation to molecular programs associated with alternative responses. MazF6 is strongly up-regulated upon overproduction of Soj_Mtb_, suggesting that the regulation of cell division and its eventual halting is facilitated through the action of this toxin. This is the first report of a coupled regulatory system that links cell cycle regulation by Soj_Mtb_ molecular programs associated with adaptation to stress in *Mtb via* the activity of MazEF6 TA loci. Importantly, this information provides a foundation for further analysis of how TA loci are coordinated with regards to other essential cellular processes and to define their contribution to the establishment of the NRP state of *Mtb*.

## Methods

### Bioinformatic analysis

To identify putative cell division regulators in *Mtb*, consensus models were derived from septum formation inhibitors from OMA groups 78690, 63437, 84083, 90270, and 245137–40 from a variety of bacterial species using a MAFFT local multialignment tool. Resulting consensus sequences were used to BLAST the *Mtb* H37Rv genome to identity mycobacterial ORFs that encode septum regulatory proteins. BLAST searches were optimized for percent identity and score. Top hits were analyzed by the Phyre2 server to confirm or refute identification as putative septum formation inhibitors.

### Generation of recombinant mycobacterial strains

For all mycobacteria experiments, *Mtb* strain H37Rv (ATCC 27294) and *M. smegmatis* mc^2^155 was cultured at 37°C with shaking in Middlebrook 7H9 liquid medium containing 0.4% glycerol, 10% OADC, and 0.05% Tween80 or on Middlebrook 7H10 agar containing 0.4% glycerol and OADC containing appropriate antibiotics as noted. For generation of recombinant mycobacterial strain overexpressing *soj*_*Mtb*_, the *rv1708* gene was PCR amplified from *Mtb* H37Rv genomic DNA using AccuPrimePfx DNA polymerase (Invitrogen) and cloned into the NdeI and HindIII of the *p*VV16 constitutive, extrachromosomal mycobacterial vector, which is a derivative of pMV261 [50,51] (Additional file 1). DNA constructs were transformed into One Shot® TOP10 chemically competent *E. coli* (Invitrogen) and selected by growth on LB agar containing 50 μg ml^-1^ kanamycin. Sequence confirmed plasmid DNA was transformed into electrocompetent *M. smegmatis* or *Mtb* and selection was carried out using kanamycin at 25 μg ml^-1^.

### Microscopy and ultrastructural analysis by SEM

Recombinant *M. smegmatis* strains containing the bacterial shuttle vector *p*VV16 as a control and *soj*_*Mtb*_::*p*VV16 were grown to an O.D._600nm_ of 1.0 and collected by centrifugation. Cells were washed three times in PBS, pH 7.4, and fixed with 2.5% glutaraldehyde in Buffer A (0.1 M potassium phosphate (pH 7.4), 1 mM CaCl_2_ and 1 mM MgCl_2_) at 4°C for 48 hrs. The fixed bacterial cells were collected by centrifugation, washed three times in Buffer A and treated with 1% OsO_4_ in Buffer A for 30 minutes at 4°C. Again, cells were washed three times with Buffer A_._ and prepared for SEM with a graded series of ethanol treatments (20-100%). Ultrastructural examination was performed using a JOEL JEM -100CX electron microscope. Recombinant *Mtb* strains containing the bacterial shuttle vector *p*VV16 as a control and *p*vv16::*soj*_*Mtb*_ were prepared as described above. However, the *p*vv16::*soj*_*Mtb*_ culture did not reach an O.D._600nm_ of 1.0 and was prepared once the growth had plateaued.

### Isolation of total RNA for microarray analysis, microarray processing, and data analysis

At Mid-Log growth, *Mtb* cells were harvested by centrifugation and resuspended in TRIzol (Invitrogen). Cells were lysed by bead beating for one-minute increments for three minutes total, cooling the sample on ice between each step. RNA was separated from other cellular products by the addition of chloroform and centrifugation, according to the TRIzol protocol. The upper aqueous layer was combined with an equal volume of 70% ethanol, and the entire volume was applied to an RNeasy Mini kit (Qiagen) column for RNA cleanup.

The *Mtb* whole genome microarrays were obtained through the TB Research Materials and Vaccine Testing Contract at Colorado State University. Slides were post-processed using succinic anhydride as described previously [4]. Approximately 8 μg of total RNA was converted to cDNA in the presence of either Cy5- or Cy3-labeled nucleotides as previously described [4,6]. Hybridization was performed at 42°C for 12 hr. Slides were scanned using an Axon Genepix scanner. The final microarray data set (Additional file 2) resulted from combining independent biological replicates. Data reduction and global normalization was performed on the raw fluorescent intensities. The normalized intensity values of treated and control cultures were used to generate ratio and log_2_ expression values for each gene.

### MazF6 interaction analysis

For the Maz6 TA loci interaction studies, *mazE and mazF* gene fragments were PCR amplified from *Mtb* H37Rv genomic DNA using Phusion® High-Fidelity PCR Master Mix with GC Buffer (New England BioLabs Inc.). Generally, *mazE* antitoxins were cloned in the BamHI and XhoI restriction sites of pET28a (Novagen), and *mazF6* toxin was cloned into the SphI and NotI restriction sites of pETcoco2 (Novagen) (Additional file 1). DNA constructs were transformed into One Shot® TOP10 chemically competent *E. coli* (Invitrogen) or Z-Competent *E. Coli* - Strain DH5α (Zymo Research) and transformants selected by growth on LB agar containing 50 μg ml^-1^ kanamycin for pET28a and 50 μg ml^-1^ ampicillin for pETcoco2. Sequence confirmed plasmid DNA was transformed into One Shot® BL21(DE3) pLysE Chemically Competent *E. coli* (Invitrogen) and selection was carried out overnight by growth in LB broth supplemented with 0.2% glucose, 34 μg ml^-1^ chloramphenicol, and containing 50 μg ml^-1^ kanamycin for pET28a selection or 50 μg ml^-1^ ampicillin for pETcoco2 selection, or both in cases of co-transformation. Glucose was added to the media to maintain low basal expression from the pET vectors as previously described [52]. The overnight outgrowths were used for recombinant protein induction. Briefly, cultures were diluted back 1:50 in fresh media containing antibiotics without glucose. When *mazF6*::pETcoco2 construct was used, diluted cultures were grown for one hour with shaking at 37°C and L-arabinose was added to a final concentration of 0.01% to amplify plasmid copy number prior to protein induction. Once cultures reached Mid-Log, protein production was induced using 1 mM final concentration of isopropyl-beta-D-thiogalactopyranoside (IPTG)(Fermentas). Cultures were incubated for four hours with shaking at 37°C, with timepoints being taken every hour to monitor growth by O.D._600nm_. After four hours, bacterial cell pellets were collected by centrifugation and stored at −80°C.

Purification of MazE-HIS in complex with MazF6-HSV was carried out under native conditions to maintain protein-protein interactions. Cleared lysates of bacterial cell pellets were obtained using BugBuster**®** including Benzonase**®** (Novagen) according to manufacturer’s instructions. Generally, cleared lysate was combined with one-fourth the volume of Ni-NTA His-Bind**®** Resin and mixed gently at 4°C for 1 hour before packing into a column. The column was washed with wash buffer 1 (500 mM NaCl, 20 mM Tris–HCl, 60 mM imidazole, pH 9.0), followed by wash buffer 2 (250 mM NaCl, 20 mM Tris–HCl, 100 mM imidazole, pH 9.0). Recombinant protein or protein complexes were eluted with elution buffer (250 mM NaCl, 20 mM Tris–HCl, 300 mM imidazole, pH 9.0). All fractions were collected and samples were resolved using pre-cast 12% NuPAGE**®** gels (Invitrogen), followed by transfer to 0.2 micron nitrocellulose membrane (BioRad) for Western blotting. Membranes were blocked in 4% BSA in TBST, incubated with primary Penta-His antibody (Qiagen) or anti-HSV antibody (Novagen), both diluted 1:10,000, followed by goat anti-mouse-alkaline phosphatase (Sigma) secondary antibody diluted 1:10,000. Membranes were developed using SIGMA*FAST*™ BCIP®/NBT tablet (Sigma) according to manufacturer’s instructions.

### Mouse infection, bacterial quantification, and RNA isolation

All use of vertebrate animals took place at Colorado State University, which is AAALAC approved and has an OLAW number of A3572-01. Care was provided by veterinarians in the Laboratory Animal Resources of Colorado State University under the supervision of the University Veterinarian. Six week-old female C57BL/6 mice (Jackson Laboratories) were infected *via* low dose aerosol with *Mtb* H37Rv in a Glas-Col Inhalation Exposure System (Glas-Col, Inc.). Exposure was conducted by aerosolizing approximately 2×10^6^ cfuml^-1^ in a volume of 5 cubic feet over a period of 30 minutes, followed by a 20 minute period of cloud decay. Infected mice were housed in sterile micro-isolator cages in the ABSL-3 facility at Colorado State University and provided water and food *ad libitum* and monitored for morbidity. Five animals per time point were sacrificed at 20, 40, and 100 days post-infection by CO_2_ inhalation and lungs and spleens were harvested. Organs were cut into halves, and half of each organ was homogenized in 2 mL sterile saline using an Omni Tissue homogenizer (Omni International). Bacterial burden in the lungs and spleen at each time point were assessed by plating 10-fold serial dilutions of organ homogenates on Middlebrook 7H11 agar containing 10% OADC and carbenicillin (Sigma) and cycloheximide (Sigma) at 50 μg ml^-1^ and 10 μg ml^-1^, respectively. Plates were incubated at 37°C for three weeks.

The remaining half of each lung and spleen were homogenized in 5 ml TRIzol by bead beating. Total RNA was isolated by organic phase separation. RNA samples were treated with DNAse (Fermentas) for 1 hour at 37°C, re-extracted in phenol/chloroform/isoamyl alcohol (25:24:1)(Sigma), and precipitated with ammonium acetate.

### Reverse transcription and quantitative-PCR

cDNA synthesis from total RNA from the lungs and spleen of infected mice was carried out using a gene-specific reverse primer cocktail with Transcriptor First Strand cDNA Synthesis Kit (Roche). The reverse primer cocktail contained reverse primers specific for *mazE6*, *mazF6*, and *soj*_*Mtb*_ in addition to random hexamers (Additional file 3). Reactions contained 1 μg total RNA, 2.5 μM gene-specific reverse primers, and 60 μM random hexamers. cDNA synthesis were carried out according to manufacturer instructions along with no reverse transcriptase control and no template control reactions. Quantative-PCR was carried out using 50 ng of cDNA generated from RNA isolated from Mid-Log phase *Mtb*, and RNA isolated from lungs and spleens of mice at Day 20, 40,and 100 post-infection. Calibration curves were generated using respective *mazE6*, *mazF6*, and *soj*_*Mtb*_ specific dsDNA amplicons generated from PCR using GoTaq®Green Master Mix (Promega). Calibration curves of respective dsDNA amplicons ranged from 1 ng to 1^-8^ ng per reaction in 10-fold serial dilutions. Modeled linear fits and primer efficiencies were used to determine concentration of respective gene. Amplicons in experimental samples were converted to total mRNA copy number per mL. Each sample was normalized by taking total mRNA copy number per mL and dividing by the associated cfu mL^-1^ recovered from each biological sample. No reverse transcriptase and no template controls confirmed no gDNA contamination or non-specific amplification.

## Competing interests

The authors declare that they have no competing interests.

## Authors’ contributions

MVR carried out the bacterial growth analysis, recombinant protein studies, interaction studies, animal studies, and isolation of total RNA and bacterial transcripts from infected tissues, CCD performed the quantitative-PCR studies from infected tissues, KE carried out the microarrays and morphological studies, and RC performed the bioinformatics. RAS designed the studies, and coordination of the manuscript. All authors participated in drafting, and editing the final manuscript. All authors have read and approved the manuscript.

## Supplementary Material

Additional file 2 **Differential gene expression of *Mtb *** **upon overexpression of *soj***_***Mtb***_**.** Click here for file

Additional file 1 Primer sequences used for plasmid construction. Click here for file

Additional file 3 Primer sequences used for end-point PCR. Click here for file
