# Supplementary material for: MazF6 toxin of Mycobacterium tuberculosis demonstrates antitoxin specificity and is coupled to regulation of cell growth by a Soj-like protein
Source: BMC Microbiol. 2013 Oct 31;13:240. doi: 10.1186/1471-2180-13-240 (PMC3834876; doi:10.1186/1471-2180-13-240)
Supplement: Additional file 2 — Differential gene expression of Mtb upon overexpression of soj Mtb . [file 1471-2180-13-240-S2.pdf]

Rv Number	Gene	Classification	Ratio change over control	Direction
RV0005	gyrB	information pathways	5.34	Down
RV0013	pabA	intermediary metabolism and respiration	2.26	Up
RV0014C	pknB	regulatory proteins	2.66	Up
RV0016C	pbpA	cell wall and cell processes	1.72	Up
RV0017C	rodA	cell wall and cell processes	1.72	Up
RV0021C		conserved hypotheticals	3.11	Up
RV0022C		conserved hypotheticals	2.83	Up
RV0024		virulence, detoxification, adaptation	2.43	Up
RV0026		conserved hypotheticals	1.55	Up
RV0028		unknown	1.68	Up
RV0030		unknown	2.55	Up
RV0032	bioF2	intermediary metabolism and respiration	2.55	Up
RV0033		lipid metabolism	3.05	Up
RV0034		unknown	1.74	Up
RV0035	fadD34	lipid metabolism	2.17	Up
RV0037C		cell wall and cell processes	2.55	Up
RV0039C		conserved hypotheticals	2.13	Up
RV0041	leuS	information pathways	2.42	Up
RV0043C		regulatory proteins	2.12	Up
RV0045C		lipid metabolism	2.62	Up
RV0046C		conserved hypotheticals	1.98	Down
RV0051		cell wall and cell processes	1.69	Up
RV0054	ssb	information pathways	2.85	Down
RV0057		unknown	2.65	Down
RV0062	celA	intermediary metabolism and respiration	2.26	Up
RV0064		cell wall and cell processes	1.58	Up
RV0068		intermediary metabolism and respiration	2.58	Up
RV0069C	sdaA	intermediary metabolism and respiration	1.7	Up
RV0070C	glyA2	intermediary metabolism and respiration	1.81	Up
RV0075		intermediary metabolism and respiration	2.04	Up
RV0076C		cell wall and cell processes	1.67	Up
RV0079		unknown	2.3	Down
RV0080		unknown	1.81	Down
RV0082		intermediary metabolism and respiration	2.95	Down
RV0083		intermediary metabolism and respiration	9.78	Down
RV0086	hycQ	intermediary metabolism and respiration	1.89	Up
RV0087	hycE	intermediary metabolism and respiration	2.82	Up
RV0089		intermediary metabolism and respiration	2.66	Up
RV0091		conserved hypotheticals	3.42	Up
RV0092	ctpA	cell wall and cell processes	2.88	Up
RV0096	PPE	PE/PPE	3.21	Up
RV0097		conserved hypotheticals	2.78	Up
RV0099	fadD10	lipid metabolism	2.28	Up
RV0108C		unknown	6.47	Down
RV0109	PE_PGRS	PE/PPE	3.09	Up
RV0111		intermediary metabolism and respiration	3.48	Up
RV0115		conserved hypotheticals	2.95	Up
RV0116C		conserved hypotheticals	1.89	Up

RV0117	oxyS	regulatory proteins	3.25	Up
RV0118C	oxcA	intermediary metabolism and respiration	1.63	Up
RV0119	fadD7	lipid metabolism	2.7	Up
RV0126		intermediary metabolism and respiration	2.1	Up
RV0133		intermediary metabolism and respiration	2.47	Up
RV0134	ephF	virulence, detoxification, adaptation	1.86	Up
RV0136		intermediary metabolism and respiration	2.67	Up
RV0137C		intermediary metabolism and respiration	3.27	Up
RV0138		conserved hypotheticals	2.62	Up
RV0140		conserved hypotheticals	2.53	Up
RV0157	pntB	intermediary metabolism and respiration	3.22	Up
RV0158		regulatory proteins	2.81	Up
RV0159C	PE	PE/PPE	3.06	Up
RV0160C	PE	PE/PPE	3.21	Up
RV0161		intermediary metabolism and respiration	3.18	Up
RV0162C	adhE	intermediary metabolism and respiration	2.66	Up
RV0180C		cell wall and cell processes	2.09	Up
RV0181C		conserved hypotheticals	3.05	Up
RV0182C	sigG	information pathways	2.99	Up
RV0187		intermediary metabolism and respiration	3.02	Up
RV0191		cell wall and cell processes	2.8	Up
RV0195		regulatory proteins	2.49	Up
RV0197		conserved hypotheticals	2.06	Up
RV0198C		intermediary metabolism and respiration	1.82	Up
RV0199		conserved hypotheticals	2.18	Up
RV0205		cell wall and cell processes	3.11	Up
RV0207C		conserved hypotheticals	2.54	Down
RV0210		unknown	2.58	Up
RV0213C		intermediary metabolism and respiration	2.76	Up
RV0218		intermediary metabolism and respiration	1.91	Up
RV0219		unknown	2.34	Up
RV0220	lipC	intermediary metabolism and respiration	1.67	Down
RV0221		conserved hypotheticals	1.99	Up
RV0223C		intermediary metabolism and respiration	2.47	Up
RV0226C		cell wall and cell processes	2.22	Up
RV0228		intermediary metabolism and respiration	3.26	Up
RV0229C		conserved hypotheticals	1.62	Up
RV0230C		conserved hypotheticals	2.3	Up
RV0235C		conserved hypotheticals	3.09	Up
RV0236C		cell wall and cell processes	2.2	Up
RV0238		regulatory proteins	2.84	Up
RV0239		conserved hypotheticals	3.19	Down
RV0250C		unknown	5.5	Down
RV0251C	hsp	virulence, detoxification, adaptation	3.47	Down
RV0253	nirD	intermediary metabolism and respiration	1.86	Up
RV0254C	cobU	intermediary metabolism and respiration	3.08	Up
RV0255C	cobQ	intermediary metabolism and respiration	2.4	Up
RV0257C		unknown	1.89	Down
RV0258C		conserved hypotheticals	3.29	Up

RV0259C		conserved hypotheticals	1.82	Up
RV0260C		conserved hypotheticals	2.17	Up
RV0261C	narK3	cell wall and cell processes	2.41	Up
RV0262C		intermediary metabolism and respiration	1.82	Up
RV0263C		conserved hypotheticals	1.67	Up
RV0265C	fecB2	cell wall and cell processes	2.05	Up
RV0267	narU	cell wall and cell processes	2.13	Up
RV0269C		conserved hypotheticals	2.33	Up
RV0271C	fadE6	lipid metabolism	2.32	Up
RV0277C		conserved hypotheticals	1.75	Up
RV0278C	PE_PGRS	PE/PPE	2.24	Up
RV0282		conserved hypotheticals	3.57	Down
RV0283		conserved hypotheticals	3.96	Down
RV0285	PE	PE/PPE	1.92	Down
RV0287		regulatory proteins	2.86	Down
RV0288		conserved hypotheticals	3.12	Down
RV0296C	atsG	intermediary metabolism and respiration	2.32	Up
RV0297	PE_PGRS	PE/PPE	3.23	Down
RV0298		unknown	3.02	Down
RV0305C	PPE	PE/PPE	2.38	Up
RV0306		intermediary metabolism and respiration	2.37	Up
RV0307C		unknown	2.49	Up
RV0311		unknown	2.94	Up
RV0313		unknown	5.83	Down
RV0317C	glpQ2	intermediary metabolism and respiration	1.76	Down
RV0318C			2.02	Up
RV0321	dcd	intermediary metabolism and respiration	2.33	Up
RV0326		unknown	2.73	Up
RV0327C		intermediary metabolism and respiration	3.97	Up
RV0328		regulatory proteins	2.1	Up
RV0329C		conserved hypotheticals	4.04	Up
RV0331		intermediary metabolism and respiration	2.6	Up
RV0335C	PE	PE/PPE	2.16	Up
RV0338C		conserved hypotheticals	2.12	Up
RV0340		unknown	1.72	Up
RV0341		conserved hypotheticals	2.89	Down
RV0344C	lpqJ	cell wall and cell processes	1.91	Up
RV0349		unknown	2.75	Up
RV0351	grpE	virulence, detoxification, adaptation	3.68	Down
RV0352	dnaJ	virulence, detoxification, adaptation	3.86	Down
RV0355C	PPE	PE/PPE	2.58	Up
RV0356C		unknown	3.29	Up
RV0357C	purA	intermediary metabolism and respiration	2.27	Up
RV0358		unknown	3.25	Up
RV0359		conserved hypotheticals	2.94	Up
RV0361		cell wall and cell processes	2.13	Up
RV0368C		conserved hypotheticals	2.86	Up
RV0371C		cell wall and cell processes	3.41	Up
RV0372C		conserved hypotheticals	2.29	Up

RV0375C		intermediary metabolism and respiration	2.72	Up
RV0376C		conserved hypotheticals	2.27	Up
RV0377		regulatory proteins	2.56	Up
RV0378		conserved hypotheticals	2.08	Up
RV0393		conserved hypotheticals	2.54	Up
RV0394C		unknown	2.32	Up
RV0395		unknown	2.57	Up
RV0396		unknown	5.24	Up
RV0398C		unknown	2.66	Up
RV0399C	lpqK	cell wall and cell processes	1.65	Up
RV0400C	fadE7	lipid metabolism	2.12	Up
RV0402C	mmpL1	cell wall and cell processes	2.55	Up
RV0406C		conserved hypotheticals	3.77	Up
RV0407		intermediary metabolism and respiration	2.33	Up
RV0408	pta	intermediary metabolism and respiration	2.49	Up
RV0413	mutT3	information pathways	4.08	Down
RV0421C		unknown	2.36	Up
RV0427C	xthA	information pathways	2.06	Up
RV0428C		unknown	2.65	Up
RV0434		conserved hypotheticals	2.53	Down
RV0442C	PPE	PE/PPE	8.92	Down
RV0446C		conserved hypotheticals	2.49	Up
RV0448C		conserved hypotheticals	1.84	Up
RV0449C		intermediary metabolism and respiration	2.75	Up
RV0452		regulatory proteins	2.64	Up
RV0453	PPE	PE/PPE	3.22	Up
RV0454		conserved hypotheticals	3.35	Up
RV0456C	echA2	lipid metabolism	3.4	Up
RV0459		conserved hypotheticals	2.95	Up
RV0462		intermediary metabolism and respiration	1.91	Up
RV0463		conserved hypotheticals	5.35	Down
RV0467	aceA	intermediary metabolism and respiration	5.04	Down
RV0471C		unknown	2.94	Up
RV0472C		regulatory proteins	1.73	Up
RV0473		cell wall and cell processes	1.84	Up
RV0480C		conserved hypotheticals	2	Up
RV0492C		intermediary metabolism and respiration	2.31	Up
RV0493C		conserved hypotheticals	3.85	Up
RV0494		regulatory proteins	1.6	Up
RV0499		unknown	3.29	Up
RV0500	proC	intermediary metabolism and respiration	2.47	Up
RV0507	mmpL2	cell wall and cell processes	2.68	Up
RV0509	hemA	intermediary metabolism and respiration	3.4	Down
RV0510	hemC	intermediary metabolism and respiration	4.69	Down
RV0513		cell wall and cell processes	2.42	Down
RV0516C		conserved hypotheticals	5.62	Down
RV0518		unknown	2.81	Up
RV0532	PE_PGRS	PE/PPE	5.23	Down
RV0535	pnp	intermediary metabolism and respiration	11.17	Down

RV0536	galE2	intermediary metabolism and respiration	6.61	Down
RV0537C		cell wall and cell processes	1.75	Up
RV0541C		cell wall and cell processes	2.31	Up
RV0543C		conserved hypotheticals	1.62	Up
RV0546C		conserved hypotheticals	3.36	Up
RV0547C		intermediary metabolism and respiration	2.95	Up
RV0548C	menB	intermediary metabolism and respiration	2.12	Up
RV0549C		conserved hypotheticals	2.08	Up
RV0550C		unknown	2.02	Up
RV0551C	fadD8	lipid metabolism	3.71	Up
RV0552		regulatory proteins	2.08	Up
RV0561C		intermediary metabolism and respiration	2.1	Up
RV0564C	gpdA1	lipid metabolism	1.69	Up
RV0565C		intermediary metabolism and respiration	3.33	Up
RV0566C		conserved hypotheticals	2.11	Up
RV0567		intermediary metabolism and respiration	2.73	Up
RV0571C		conserved hypotheticals	3.04	Up
RV0573C		conserved hypotheticals	3.15	Up
RV0574C		conserved hypotheticals	2.68	Up
RV0575C		intermediary metabolism and respiration	2.27	Up
RV0578C	PE_PGRS	PE/PPE	8.12	Down
RV0585C		cell wall and cell processes	3.12	Up
RV0588		virulence, detoxification, adaptation	1.5	Up
RV0590		virulence, detoxification, adaptation	1.99	Up
RV0592		virulence, detoxification, adaptation	2.83	Up
RV0595C		conserved hypotheticals	3.34	Up
RV0596C		conserved hypotheticals	2.3	Up
RV0597C		conserved hypotheticals	2.82	Up
RV0600C		regulatory proteins	3.42	Up
RV0601C		regulatory proteins	2.13	Up
RV0603		unknown	2.83	Up
RV0604	lpqO	cell wall and cell processes	2.96	Up
RV0614		conserved hypotheticals	2.93	Up
RV0615		cell wall and cell processes	2.22	Down
RV0616C		unknown	2.09	Up
RV0618	galT'	intermediary metabolism and respiration	2.76	Up
RV0619	galT	intermediary metabolism and respiration	2.97	Up
RV0620	galK	intermediary metabolism and respiration	2.76	Up
RV0621		cell wall and cell processes	2.87	Up
RV0622		cell wall and cell processes	3.54	Up
RV0623		conserved hypotheticals	2.31	Up
RV0625C		cell wall and cell processes	2.38	Up
RV0630C	recB	information pathways	2.91	Up
RV0638	secE	cell wall and cell processes	3.51	Down
RV0639	nusG	information pathways	2.16	Down
RV0641	rplA	information pathways	2.66	Down
RV0645C	mmaA1	lipid metabolism	3.38	Up
RV0647C		conserved hypotheticals	2.65	Up
RV0648		conserved hypotheticals	2.47	Up

RV0650		regulatory proteins	3.41	Up
RV0651	rplJ	information pathways	3.33	Up
RV0652	rplL	information pathways	1.83	Up
RV0653C		regulatory proteins	2.7	Up
RV0655		cell wall and cell processes	1.79	Down
RV0658C		cell wall and cell processes	1.82	Up
RV0660C		conserved hypotheticals	4.34	Down
RV0661C		conserved hypotheticals	3.16	Up
RV0663	atsD	intermediary metabolism and respiration	3.14	Up
RV0665		conserved hypotheticals	2.57	Up
RV0667	rpoB	information pathways	2.48	Down
RV0668	rpoC	information pathways	1.84	Down
RV0669C		conserved hypotheticals	2.41	Up
RV0671	lpqP	cell wall and cell processes	1.87	Up
RV0688		intermediary metabolism and respiration	2.48	Up
RV0692		unknown	4.86	Down
RV0702	rplD	information pathways	3.15	Down
RV0706	rplV	information pathways	3.73	Down
RV0707	rpsC	information pathways	7.43	Down
RV0712		conserved hypotheticals	2.31	Up
RV0713		cell wall and cell processes	2.47	Up
RV0722	rpmD	information pathways	2.02	Down
RV0723	rplO	information pathways	1.99	Down
RV0727C	fucA	intermediary metabolism and respiration	2.49	Up
RV0731C		conserved hypotheticals	2.61	Up
RV0732	secY	cell wall and cell processes	2.26	Up
RV0735	sigL	information pathways	2.93	Up
RV0737		regulatory proteins	2.81	Up
RV0739		conserved hypotheticals	2.42	Up
RV0740		conserved hypotheticals	2.05	Up
RV0741		insertion seqs and phages	2.27	Up
RV0742	PE_PGRS	PE/PPE	5.96	Down
RV0743C		unknown	2.41	Up
RV0746	PE_PGRS	PE/PPE	2.54	Up
RV0748		conserved hypotheticals	3.94	Down
RV0761C	adhB	intermediary metabolism and respiration	3.08	Down
RV0762C		unknown	3.86	Up
RV0764C		intermediary metabolism and respiration	2.76	Up
RV0765C		intermediary metabolism and respiration	2.66	Up
RV0766C		intermediary metabolism and respiration	3.35	Up
RV0767C		unknown	2.93	Up
RV0768	aldA	intermediary metabolism and respiration	2.94	Up
RV0769		intermediary metabolism and respiration	2.22	Up
RV0772	purD	intermediary metabolism and respiration	2.13	Up
RV0779C		conserved hypotheticals	2.52	Up
RV0781	ptrBb	intermediary metabolism and respiration	2.18	Up
RV0782	ptrBa	intermediary metabolism and respiration	3.49	Up
RV0783C		cell wall and cell processes	3.5	Up
RV0784		conserved hypotheticals	2.76	Up

RV0794C	lpdB	intermediary metabolism and respiration	2.07	Up
RV0797		insertion seqs and phages	2.89	Up
RV0807		conserved hypotheticals	2.21	Up
RV0811C		conserved hypotheticals	2.01	Up
RV0812	pabC	intermediary metabolism and respiration	2.35	Up
RV0813C		conserved hypotheticals	1.96	Up
RV0816C	thiX	intermediary metabolism and respiration	2.35	Up
RV0817C		cell wall and cell processes	2.55	Up
RV0820	phoT	cell wall and cell processes	1.9	Up
RV0823C		regulatory proteins	4.28	Down
RV0824C	desA1	lipid metabolism	22.48	Down
RV0830		conserved hypotheticals	2.66	Up
RV0838	lpqR	cell wall and cell processes	2.11	Up
RV0845		regulatory proteins	2.15	Up
RV0849		cell wall and cell processes	2.07	Up
RV0850		insertion seqs and phages	2.29	Up
RV0858C		intermediary metabolism and respiration	2.89	Up
RV0872C	PE_PGRS	PE/PPE	14.79	Down
RV0882		cell wall and cell processes	2.19	Up
RV0883C		conserved hypotheticals	2.56	Up
RV0884C	serC	intermediary metabolism and respiration	2.02	Up
RV0886	fprB	intermediary metabolism and respiration	2.15	Up
RV0888		cell wall and cell processes	1.64	Up
RV0894		regulatory proteins	2.29	Up
RV0901		conserved hypotheticals	2.34	Up
RV0902C		regulatory proteins	1.92	Up
RV0903C		regulatory proteins	1.76	Up
RV0905	echA6	lipid metabolism	2.06	Up
RV0906		cell wall and cell processes	2	Up
RV0907		cell wall and cell processes	2.99	Up
RV0908	ctpE	cell wall and cell processes	2.2	Up
RV0912		conserved hypotheticals	2.07	Up
RV0920C		insertion seqs and phages	2.08	Up
RV0925C		unknown	1.87	Up
RV0927C		intermediary metabolism and respiration	2.41	Up
RV0928	phoS2	cell wall and cell processes	3	Up
RV0929	pstC2	cell wall and cell processes	3.04	Up
RV0930	pstA1	cell wall and cell processes	3.03	Up
RV0932C	pstS	cell wall and cell processes	1.86	Down
RV0936	pstA2	cell wall and cell processes	2.35	Up
RV0942		unknown	2.22	Up
RV0957	purH	intermediary metabolism and respiration	2.04	Up
RV0963C		conserved hypotheticals	2.97	Up
RV0964C		unknown	1.8	Up
RV0965C		conserved hypotheticals	3.3	Up
RV0966C		conserved hypotheticals	2.54	Up
RV0968		conserved hypotheticals	2	Up
RV0973C	accA2	lipid metabolism	2.35	Up
RV0975C	fadE13	lipid metabolism	4.15	Up

RV0976C		unknown	3.25	Up
RV0977	PE_PGRS	PE/PPE	3.15	Up
RV0978C	PE_PGRS	PE/PPE	3.81	Up
RV0979C		unknown	4.56	Up
RV0980C	PE_PGRS	PE/PPE	3.74	Up
RV0982		regulatory proteins	3.9	Up
RV0983		intermediary metabolism and respiration	4.07	Up
RV0984	moaB2	intermediary metabolism and respiration	3.25	Up
RV0985C	mscL	cell wall and cell processes	2.66	Up
RV0986		cell wall and cell processes	3.75	Up
RV0987		cell wall and cell processes	3.56	Up
RV0988		conserved hypotheticals	3.04	Up
RV0990C		unknown	3.04	Up
RV0992C		conserved hypotheticals	1.93	Up
RV1012		unknown	2.3	Up
RV1013	pks16	lipid metabolism	2.25	Down
RV1016C	lpqT	cell wall and cell processes	2.16	Up
RV1022	lpqU	cell wall and cell processes	3.12	Up
RV1023	eno	intermediary metabolism and respiration	2.18	Up
RV1024		unknown	1.76	Up
RV1025		unknown	2.71	Up
RV1026		virulence, detoxification, adaptation	2.72	Up
RV1027C	kdpE	regulatory proteins	3.33	Up
RV1030	kdpB	cell wall and cell processes	2.67	Up
RV1032C		regulatory proteins	2.63	Up
RV1037C		conserved hypotheticals	23.98	Down
RV1038C		conserved hypotheticals	40.12	Down
RV1042C		insertion seqs and phages	5.84	Down
RV1045		unknown	2.06	Up
RV1046C		unknown	5.5	Down
RV1049		regulatory proteins	2.61	Up
RV1051C		conserved hypotheticals	3.21	Up
RV1052		unknown	2.13	Up
RV1053C		unknown	3.45	Up
RV1055		insertion seqs and phages	2.34	Up
RV1056		conserved hypotheticals	2.03	Up
RV1058	fadD14	lipid metabolism	2.52	Up
RV1065		conserved hypotheticals	5.63	Down
RV1069C		conserved hypotheticals	3.36	Up
RV1072		cell wall and cell processes	4.79	Down
RV1075C		conserved hypotheticals	2.77	Up
RV1076	lipU	intermediary metabolism and respiration	2.59	Down
RV1079	metB	intermediary metabolism and respiration	2.44	Up
RV1080C	greA	information pathways	2.16	Down
RV1090		intermediary metabolism and respiration	1.5	Up
RV1091	PE_PGRS	PE/PPE	2.12	Down
RV1094	desA2	lipid metabolism	7.23	Down
RV1099C		conserved hypotheticals	2.74	Up
RV1105		intermediary metabolism and respiration	1.74	Up

RV1107C	xseB	intermediary metabolism and respiration	2.92	Down
RV1109C		unknown	4.9	Down
RV1110	lytB'	cell wall and cell processes	5.59	Down
RV1118C		unknown	2.66	Up
RV1119C		unknown	3.21	Up
RV1120C		conserved hypotheticals	3.07	Up
RV1121	zwf	intermediary metabolism and respiration	2.05	Up
RV1123C	bpoB	virulence, detoxification, adaptation	3.63	Up
RV1124	ephC	virulence, detoxification, adaptation	3.08	Up
RV1125		unknown	3.55	Up
RV1128C		insertion seqs and phages	2.08	Up
RV1130		conserved hypotheticals	2.83	Down
RV1131	gltA1	intermediary metabolism and respiration	5.86	Down
RV1132		cell wall and cell processes	1.7	Up
RV1133C	metE	intermediary metabolism and respiration	10.33	Down
RV1135C	PPE	PE/PPE	9.42	Down
RV1136		lipid metabolism	1.9	Up
RV1137C		unknown	1.86	Up
RV1138C		conserved hypotheticals	1.83	Up
RV1141C	echA11	lipid metabolism	2.64	Up
RV1143	mcr	lipid metabolism	2.52	Up
RV1144		intermediary metabolism and respiration	1.84	Up
RV1145		cell wall and cell processes	3.64	Up
RV1146		cell wall and cell processes	3.38	Up
RV1147		intermediary metabolism and respiration	2.5	Up
RV1148C		insertion seqs and phages	3.34	Up
RV1153C	omt	lipid metabolism	2.3	Up
RV1155		unknown	1.67	Down
RV1156		unknown	2.66	Down
RV1157C		conserved hypotheticals	2.15	Down
RV1158C		conserved hypotheticals	2.31	Down
RV1165		conserved hypotheticals	2.19	Up
RV1166	lpqW	cell wall and cell processes	2.08	Up
RV1171		unknown	2.5	Up
RV1174C		unknown	4.15	Down
RV1175C	fadH	lipid metabolism	2.04	Up
RV1176C		conserved hypotheticals	1.78	Up
RV1177	fdxC	intermediary metabolism and respiration	13.81	Down
RV1178		intermediary metabolism and respiration	4.8	Down
RV1179C		unknown	2.08	Down
RV1184C		conserved hypotheticals	3.48	Down
RV1185C	fadD21	lipid metabolism	3.95	Down
RV1189	sigI	information pathways	2.35	Up
RV1190		conserved hypotheticals	2.41	Up
RV1195	PE	PE/PPE	11.36	Down
RV1196	PPE	PE/PPE	13.43	Down
RV1197		conserved hypotheticals	17.97	Down
RV1198		conserved hypotheticals	9.73	Down
RV1208		conserved hypotheticals	2.21	Down

RV1209		conserved hypotheticals	2.92	Down
RV1212C		cell wall and cell processes	1.58	Up
RV1216C		conserved hypotheticals	2.17	Up
RV1220C		intermediary metabolism and respiration	1.86	Up
RV1226C		cell wall and cell processes	4.85	Down
RV1236	sugA	cell wall and cell processes	2.25	Up
RV1237	sugB	cell wall and cell processes	2.35	Up
RV1238	sugC	cell wall and cell processes	1.97	Up
RV1241		conserved hypotheticals	2.06	Up
RV1243C	PE_PGRS	PE/PPE	3.28	Down
RV1244	lpqZ	cell wall and cell processes	2.17	Up
RV1246C		conserved hypotheticals	2.25	Up
RV1248C	sucA	intermediary metabolism and respiration	1.66	Up
RV1261C		conserved hypotheticals	1.96	Up
RV1263	amiB2	intermediary metabolism and respiration	2.9	Up
RV1264		intermediary metabolism and respiration	2.13	Up
RV1267C	embR	regulatory proteins	2.47	Up
RV1268C		unknown	2.78	Up
RV1269C		conserved hypotheticals	2.3	Up
RV1272C		cell wall and cell processes	2.81	Up
RV1286	cysN	intermediary metabolism and respiration	1.64	Up
RV1289		unknown	2.22	Up
RV1292	argS	information pathways	2.67	Up
RV1297	rho	information pathways	7.26	Down
RV1300	hemK	intermediary metabolism and respiration	2.72	Down
RV1304	atpB	intermediary metabolism and respiration	14.58	Down
RV1306	atpF	intermediary metabolism and respiration	9.66	Down
RV1307	atpH	intermediary metabolism and respiration	5.08	Down
RV1308	atpA	intermediary metabolism and respiration	7.68	Down
RV1311	atpC	intermediary metabolism and respiration	2.96	Down
RV1314C		conserved hypotheticals	2.05	Up
RV1316C	ogt	information pathways	2.14	Up
RV1318C		intermediary metabolism and respiration	2.06	Up
RV1319C		intermediary metabolism and respiration	1.83	Up
RV1320C		intermediary metabolism and respiration	2.25	Up
RV1327C		intermediary metabolism and respiration	4.56	Down
RV1330C		conserved hypotheticals	2.42	Up
RV1339		conserved hypotheticals	1.91	Up
RV1341		conserved hypotheticals	1.88	Up
RV1342C	pks14	lipid metabolism	2.71	Up
RV1349		cell wall and cell processes	2.09	Up
RV1350	fabG2	lipid metabolism	2.81	Up
RV1353C		regulatory proteins	3.73	Up
RV1354C		conserved hypotheticals	1.93	Up
RV1357C		conserved hypotheticals	3.42	Up
RV1359		regulatory proteins	2.95	Up
RV1366		unknown	2.79	Up
RV1369C		insertion seqs and phages	2.94	Up
RV1371		unknown	2.49	Up

RV1374C		unknown	2.53	Up
RV1376		conserved hypotheticals	1.71	Up
RV1379	pyrR	regulatory proteins	2.74	Up
RV1381	pyrC	intermediary metabolism and respiration	3.11	Up
RV1385	pyrF	intermediary metabolism and respiration	2.33	Up
RV1386	PE	PE/PPE	3.41	Down
RV1387	PPE	PE/PPE	4.58	Down
RV1389	gmk	intermediary metabolism and respiration	1.74	Down
RV1395		regulatory proteins	2.29	Up
RV1396C	PE_PGRS	PE/PPE	10.59	Down
RV1397C		conserved hypotheticals	3.87	Down
RV1398C		conserved hypotheticals	8.08	Down
RV1404		regulatory proteins	3.04	Down
RV1405C		intermediary metabolism and respiration	1.83	Up
RV1411C	lprG	cell wall and cell processes	1.57	Down
RV1412	ribC	intermediary metabolism and respiration	2.33	Up
RV1413		unknown	2.28	Up
RV1414		unknown	2.98	Up
RV1421		conserved hypotheticals	2.57	Up
RV1425		conserved hypotheticals	1.75	Up
RV1427C	fadD12	lipid metabolism	3.06	Up
RV1429		conserved hypotheticals	1.8	Up
RV1430	PE	PE/PPE	2.69	Up
RV1434		unknown	2.54	Up
RV1439C		unknown	2.31	Up
RV1443C		unknown	1.53	Up
RV1445C	devB	intermediary metabolism and respiration	1.67	Up
RV1452C	PE_PGRS	PE/PPE	10.74	Down
RV1455		conserved hypotheticals	3.31	Up
RV1457C		cell wall and cell processes	3.72	Up
RV1458C		cell wall and cell processes	2.19	Up
RV1469	ctpD	cell wall and cell processes	1.72	Up
RV1470	trxA	intermediary metabolism and respiration	1.7	Up
RV1475C	acn	intermediary metabolism and respiration	2.51	Down
RV1476		unknown	1.61	Up
RV1482C		conserved hypotheticals	2.66	Up
RV1485	hemZ	intermediary metabolism and respiration	3.31	Up
RV1490		cell wall and cell processes	2.21	Up
RV1492	mutA	lipid metabolism	2.07	Up
RV1493	mutB	lipid metabolism	1.77	Up
RV1499		unknown	1.66	Up
RV1501		conserved hypotheticals	2.4	Up
RV1503C		conserved hypotheticals	2.85	Up
RV1504C		conserved hypotheticals	2.49	Up
RV1505C		conserved hypotheticals	3.28	Up
RV1506C		unknown	3.07	Up
RV1507C		unknown	2.87	Up
RV1508C		cell wall and cell processes	2.08	Up
RV1509		unknown	2.55	Up

RV1510		cell wall and cell processes	2.95	Up
RV1511	gmdA	intermediary metabolism and respiration	2.58	Up
RV1512	epiA	intermediary metabolism and respiration	2.81	Up
RV1515C		conserved hypotheticals	2.12	Up
RV1517		conserved hypotheticals	1.92	Up
RV1518		cell wall and cell processes	1.83	Down
RV1524		intermediary metabolism and respiration	1.56	Up
RV1528C	papA4	lipid metabolism	3.35	Up
RV1529	fadD24	lipid metabolism	4.01	Up
RV1530	adh	intermediary metabolism and respiration	2.71	Up
RV1543		lipid metabolism	3.09	Down
RV1550	fadD11	lipid metabolism	3.04	Up
RV1552	frdA	intermediary metabolism and respiration	2.39	Up
RV1553	frdB	intermediary metabolism and respiration	3.23	Up
RV1554	frdC	intermediary metabolism and respiration	2.74	Up
RV1556		regulatory proteins	2.65	Up
RV1571		conserved hypotheticals	4.72	Down
RV1573		insertion seqs and phages	2.67	Up
RV1576C		insertion seqs and phages	1.71	Up
RV1577C		insertion seqs and phages	2.58	Up
RV1585C		insertion seqs and phages	1.77	Down
RV1593C		conserved hypotheticals	2.33	Down
RV1594	nadA	intermediary metabolism and respiration	10.46	Down
RV1595	nadB	intermediary metabolism and respiration	7.46	Down
RV1597		unknown	1.94	Up
RV1600	hisC	intermediary metabolism and respiration	1.61	Up
RV1601	hisB	intermediary metabolism and respiration	1.9	Up
RV1602	hisH	intermediary metabolism and respiration	1.72	Up
RV1606	hisI2	intermediary metabolism and respiration	1.8	Up
RV1607	chaA	cell wall and cell processes	2.53	Up
RV1613	trpA	intermediary metabolism and respiration	2.78	Down
RV1619		conserved hypotheticals	1.71	Up
RV1620C	cydC	intermediary metabolism and respiration	3.43	Down
RV1625C		intermediary metabolism and respiration	2.43	Up
RV1629	polA	information pathways	1.98	Up
RV1632C		unknown	1.65	Up
RV1639C		conserved hypotheticals	5.86	Down
RV1642	rpmI	information pathways	10.61	Down
RV1643	rplT	information pathways	7.79	Down
RV1647		conserved hypotheticals	2.06	Up
RV1651C	PE_PGRS	PE/PPE	2.21	Up
RV1652	argC	intermediary metabolism and respiration	2.63	Up
RV1656	argF	intermediary metabolism and respiration	2.35	Up
RV1674C		regulatory proteins	1.99	Up
RV1680		unknown	1.58	Up
RV1684		conserved hypotheticals	2.41	Up
RV1685C		conserved hypotheticals	2.3	Up
RV1694	tlyA	virulence, detoxification, adaptation	1.61	Up
RV1695		conserved hypotheticals	2.57	Up

RV1696	recN	information pathways	2.56	Up
RV1699	pyrG	intermediary metabolism and respiration	2.2	Up
RV1702C		insertion seqs and phages	2.35	Up
RV1717		conserved hypotheticals	2.29	Up
RV1718		conserved hypotheticals	2.22	Up
RV1719		regulatory proteins	2.01	Up
RV1720C		conserved hypotheticals	2.74	Up
RV1721C		unknown	2.13	Up
RV1727		conserved hypotheticals	2.43	Up
RV1729C		conserved hypotheticals	3.54	Up
RV1738		conserved hypotheticals	6.74	Down
RV1741		conserved hypotheticals	2.09	Up
RV1745C		conserved hypotheticals	3.07	Up
RV1746	pknF	regulatory proteins	2.48	Up
RV1747		cell wall and cell processes	2.12	Up
RV1748		unknown	2.16	Down
RV1749C		cell wall and cell processes	2.94	Up
RV1751		intermediary metabolism and respiration	2.94	Up
RV1753C	PPE	PE/PPE	1.9	Up
RV1755C	plcD	intermediary metabolism and respiration	3.3	Up
RV1760		conserved hypotheticals	1.91	Up
RV1761C		unknown	2.44	Up
RV1767		conserved hypotheticals	2.38	Up
RV1769		unknown	3.45	Up
RV1773C		regulatory proteins	1.97	Up
RV1774		intermediary metabolism and respiration	1.96	Up
RV1776C		regulatory proteins	2.39	Up
RV1779C		cell wall and cell processes	2.23	Up
RV1786		intermediary metabolism and respiration	1.91	Up
RV1792		conserved hypotheticals	29.36	Down
RV1793		conserved hypotheticals	12.37	Down
RV1796		conserved hypotheticals	2.79	Down
RV1800	PPE	PE/PPE	2.74	Up
RV1801	PPE	PE/PPE	3.37	Up
RV1802	PPE	PE/PPE	2.72	Up
RV1803C	PE_PGRS	PE/PPE	3.53	Up
RV1806	PE	PE/PPE	1.92	Up
RV1807	PPE	PE/PPE	3.2	Up
RV1814		lipid metabolism	2.85	Up
RV1816		regulatory proteins	2.43	Up
RV1817		intermediary metabolism and respiration	2.6	Up
RV1818C	PE_PGRS	PE/PPE	1.96	Down
RV1819C		cell wall and cell processes	2.62	Up
RV1823		conserved hypotheticals	3.06	Up
RV1824		cell wall and cell processes	2.46	Up
RV1827		conserved hypotheticals	5.49	Down
RV1828		conserved hypotheticals	2.76	Down
RV1829		conserved hypotheticals	2.08	Down
RV1831		unknown	4.58	Down

RV1837C	glcB	intermediary metabolism and respiration	2.58	Down
RV1839C		conserved hypotheticals	2.04	Up
RV1842C		cell wall and cell processes	4.46	Down
RV1846C		regulatory proteins	3.24	Down
RV1852	ureG	intermediary metabolism and respiration	2.04	Up
RV1853	ureD	intermediary metabolism and respiration	2.19	Down
RV1854C	ndh	intermediary metabolism and respiration	2.45	Up
RV1861		unknown	2.49	Up
RV1862	adhA	intermediary metabolism and respiration	2.41	Up
RV1864C		conserved hypotheticals	2.61	Up
RV1865C		intermediary metabolism and respiration	3.08	Up
RV1866		conserved hypotheticals	3.72	Up
RV1867		lipid metabolism	3.34	Up
RV1868		conserved hypotheticals	2.51	Up
RV1870C		unknown	4.14	Down
RV1871C		unknown	5.87	Down
RV1872C	lldD2	intermediary metabolism and respiration	7.22	Down
RV1879		conserved hypotheticals	2.32	Up
RV1884C		conserved hypotheticals	3.58	Down
RV1889C		conserved hypotheticals	2.3	Up
RV1890C		unknown	1.97	Up
RV1895		intermediary metabolism and respiration	3.05	Up
RV1896C		conserved hypotheticals	2.62	Up
RV1899C	lppD	cell wall and cell processes	2.33	Up
RV1902C	nanT	cell wall and cell processes	2.94	Up
RV1903		cell wall and cell processes	2.6	Up
RV1910C		cell wall and cell processes	1.78	Up
RV1912C	fadB5	lipid metabolism	2.15	Up
RV1913		unknown	3.65	Up
RV1914C		unknown	2.04	Up
RV1917C	PPE	PE/PPE	3.91	Up
RV1925	fadD31	lipid metabolism	8.25	Down
RV1927		unknown	8.13	Down
RV1932	tpx	virulence, detoxification, adaptation	2.67	Down
RV1933C	fadE18	lipid metabolism	3.11	Up
RV1936		intermediary metabolism and respiration	3.32	Up
RV1939		intermediary metabolism and respiration	2.65	Up
RV1940	ribA	intermediary metabolism and respiration	1.89	Up
RV1941		intermediary metabolism and respiration	2.66	Up
RV1955		unknown	3.57	Down
RV1956		regulatory proteins	2.59	Down
RV1957		unknown	5.26	Down
RV1958C		unknown	1.62	Up
RV1961		unknown	3.02	Up
RV1963C		regulatory proteins	2.68	Up
RV1966	mce3	virulence, detoxification, adaptation	3.14	Up
RV1967		virulence, detoxification, adaptation	1.81	Up
RV1968		virulence, detoxification, adaptation	3.46	Up
RV1970	lprM	cell wall and cell processes	2.23	Up

RV1971		virulence, detoxification, adaptation	1.83	Up
RV1980C	mpt64	cell wall and cell processes	1.78	Down
RV1981C	nrdF	intermediary metabolism and respiration	3.7	Up
RV1983	PE_PGRS	PE/PPE	3.24	Up
RV1984C		cell wall and cell processes	1.78	Up
RV1985C		regulatory proteins	2.78	Up
RV1986		cell wall and cell processes	1.98	Up
RV1987		cell wall and cell processes	2.77	Down
RV1988		intermediary metabolism and respiration	2.68	Up
RV1991C		conserved hypotheticals	20.72	Up
RV1993C		conserved hypotheticals	3.31	Down
RV1999C		cell wall and cell processes	2.04	Up
RV2000		unknown	1.6	Up
RV2004C		unknown	7.58	Down
RV2007C	fdxA	intermediary metabolism and respiration	4.01	Down
RV2010		conserved hypotheticals	2.06	Down
RV2012		unknown	2.14	Up
RV2014		insertion seqs and phages	1.71	Up
RV2020C		unknown	2.55	Down
RV2023C		unknown	3.54	Down
RV2028C		conserved hypotheticals	4.59	Down
RV2029C	pfkB	intermediary metabolism and respiration	4.4	Down
RV2030C		conserved hypotheticals	5.01	Down
RV2031C	hspX	virulence, detoxification, adaptation	8.07	Down
RV2038C		cell wall and cell processes	2.58	Up
RV2040C		cell wall and cell processes	2.41	Up
RV2053C		conserved hypotheticals	2.34	Down
RV2058C	rpmB2	information pathways	1.96	Up
RV2059		conserved hypotheticals	2.24	Up
RV2060		conserved hypotheticals	2.79	Up
RV2062C	cobN	intermediary metabolism and respiration	1.79	Up
RV2064	cobG	intermediary metabolism and respiration	2.38	Up
RV2071C	cobM	intermediary metabolism and respiration	2.12	Up
RV2072C	cobL	intermediary metabolism and respiration	1.82	Up
RV2077C		conserved hypotheticals	4.44	Down
RV2078		unknown	3.8	Up
RV2080	lppJ	cell wall and cell processes	2.45	Up
RV2084		unknown	2.14	Up
RV2085		insertion seqs and phages	2.86	Up
RV2088	pknJ	regulatory proteins	3.26	Up
RV2089C	pepE	intermediary metabolism and respiration	1.99	Up
RV2091C		cell wall and cell processes	3.26	Down
RV2094C		conserved hypotheticals	8.07	Down
RV2106		insertion seqs and phages	3.26	Up
RV2108	PPE	PE/PPE	3.06	Up
RV2109C	prcA	intermediary metabolism and respiration	1.85	Up
RV2121C	hisG	intermediary metabolism and respiration	3.83	Up
RV2126C	PE_PGRS	PE/PPE	7.32	Down
RV2128		unknown	1.66	Up

RV2129C		intermediary metabolism and respiration	2.21	Up
RV2132		conserved hypotheticals	1.89	Up
RV2134C		conserved hypotheticals	2.41	Down
RV2135C		conserved hypotheticals	2	Up
RV2143		conserved hypotheticals	3.04	Up
RV2147C		unknown	2.24	Up
RV2159C		unknown	2.71	Down
RV2160C		unknown	9.55	Down
RV2161C		intermediary metabolism and respiration	3.41	Down
RV2162C	PE_PGRS	PE/PPE	6.93	Down
RV2163C	pbpB	cell wall and cell processes	1.6	Up
RV2164C		unknown	1.72	Down
RV2166C		conserved hypotheticals	3.58	Up
RV2167C		insertion seqs and phages	3.43	Up
RV2172C		unknown	2.29	Down
RV2178C	aroG	intermediary metabolism and respiration	1.95	Up
RV2179C		unknown	3.19	Up
RV2180C		cell wall and cell processes	3.71	Up
RV2183C		unknown	2.11	Up
RV2193	ctaE	intermediary metabolism and respiration	1.52	Down
RV2201	asnB	intermediary metabolism and respiration	2.58	Up
RV2203		unknown	2.63	Up
RV2204C		conserved hypotheticals	2.85	Down
RV2207	cobT	intermediary metabolism and respiration	2.49	Up
RV2208	cobS	intermediary metabolism and respiration	1.99	Up
RV2211C	gcvT	intermediary metabolism and respiration	2.24	Up
RV2223C		cell wall and cell processes	2.17	Up
RV2225	panB	intermediary metabolism and respiration	2.43	Up
RV2226		unknown	2.34	Up
RV2227		unknown	1.71	Up
RV2228C		unknown	2.06	Up
RV2230C		conserved hypotheticals	2.99	Up
RV2231C	cobC	intermediary metabolism and respiration	1.52	Up
RV2232		unknown	3.27	Up
RV2235		unknown	1.66	Up
RV2236C	cobD	intermediary metabolism and respiration	2.21	Up
RV2240C		conserved hypotheticals	2.55	Up
RV2241	aceE	intermediary metabolism and respiration	4.31	Down
RV2242		unknown	2.64	Up
RV2244	acpM	lipid metabolism	3.65	Down
RV2245	kasA	lipid metabolism	3.64	Down
RV2246	kasB	lipid metabolism	7.47	Down
RV2248		conserved hypotheticals	2.78	Up
RV2252		conserved hypotheticals	3.22	Up
RV2254C		unknown	2.4	Up
RV2256C		conserved hypotheticals	2.99	Down
RV2265		cell wall and cell processes	2.47	Up
RV2267C		unknown	2.41	Up
RV2269C		unknown	3.23	Up

RV2274C		unknown	3.14	Up
RV2275		unknown	3.57	Up
RV2277C		intermediary metabolism and respiration	4.22	Up
RV2279		insertion seqs and phages	3.31	Up
RV2282C		regulatory proteins	2.78	Up
RV2287	yjcE	cell wall and cell processes	10.38	Down
RV2293C		unknown	2.68	Up
RV2294		intermediary metabolism and respiration	2.91	Up
RV2296		intermediary metabolism and respiration	2.23	Up
RV2299C	htpG	virulence, detoxification, adaptation	2.14	Up
RV2300C		conserved hypotheticals	3.04	Up
RV2301		cell wall and cell processes	1.74	Up
RV2303C		intermediary metabolism and respiration	2.63	Up
RV2304C		unknown	2.4	Up
RV2309C		insertion seqs and phages	1.68	Down
RV2317	uspE	cell wall and cell processes	3.71	Up
RV2318	uspC	cell wall and cell processes	3.1	Up
RV2319C		unknown	3.59	Up
RV2320C	rocE	cell wall and cell processes	2.63	Up
RV2321C	rocD2	intermediary metabolism and respiration	3.49	Up
RV2322C	rocD1	intermediary metabolism and respiration	2.79	Up
RV2323C		conserved hypotheticals	2.61	Up
RV2324		regulatory proteins	2.22	Up
RV2329C	narK1	cell wall and cell processes	2.01	Down
RV2330C	lppP	cell wall and cell processes	2.5	Up
RV2335	cysE	intermediary metabolism and respiration	1.74	Down
RV2337C		unknown	1.5	Up
RV2340C	PE	PE/PPE	1.98	Up
RV2341	lppQ	cell wall and cell processes	3.26	Up
RV2343C	dnaG	information pathways	3.15	Up
RV2346C		conserved hypotheticals	10.53	Down
RV2347C		conserved hypotheticals	13.12	Down
RV2348C		unknown	6.96	Down
RV2349C	plcC	intermediary metabolism and respiration	1.78	Up
RV2350C	plcB	intermediary metabolism and respiration	1.86	Up
RV2351C	plcA	intermediary metabolism and respiration	3.03	Up
RV2355		insertion seqs and phages	2.71	Up
RV2356C	PPE	PE/PPE	1.88	Up
RV2368C	phoH	intermediary metabolism and respiration	1.55	Up
RV2369C		unknown	2.33	Down
RV2370C		conserved hypotheticals	3.22	Up
RV2371	PE	PE/PPE	2.82	Down
RV2384	mbtA	lipid metabolism	2.03	Up
RV2385	lipK	intermediary metabolism and respiration	2.29	Up
RV2390C		conserved hypotheticals	1.82	Up
RV2391	nirA	intermediary metabolism and respiration	2.48	Down
RV2392	cysH	intermediary metabolism and respiration	2.24	Down
RV2394	ggtB	intermediary metabolism and respiration	2.42	Up
RV2395		cell wall and cell processes	2.35	Up

RV2396	PE_PGRS	PE/PPE	1.89	Down
RV2398C	cysW	cell wall and cell processes	2.36	Up
RV2406C		conserved hypotheticals	2.34	Down
RV2408	PE	PE/PPE	2.25	Up
RV2413C		conserved hypotheticals	1.89	Up
RV2415C		conserved hypotheticals	3.58	Up
RV2419C		intermediary metabolism and respiration	1.83	Up
RV2420C		conserved hypotheticals	2.58	Up
RV2421C		conserved hypotheticals	2.35	Up
RV2423		unknown	2.88	Up
RV2424C		insertion seqs and phages	2.37	Up
RV2428	ahpC	virulence, detoxification, adaptation	5.98	Up
RV2432C		unknown	3.55	Down
RV2439C	proB	intermediary metabolism and respiration	2.93	Up
RV2440C	obg	intermediary metabolism and respiration	1.78	Down
RV2442C	rplU	information pathways	1.91	Down
RV2444C	rne	intermediary metabolism and respiration	4.77	Down
RV2445C	ndkA	intermediary metabolism and respiration	1.89	Up
RV2446C		unknown	5.02	Down
RV2447C	folC	intermediary metabolism and respiration	2.28	Up
RV2451		unknown	2	Up
RV2452C		unknown	2.03	Up
RV2457C	clpX	intermediary metabolism and respiration	2.48	Down
RV2461C	clpP	intermediary metabolism and respiration	1.51	Down
RV2467	pepD	intermediary metabolism and respiration	1.91	Up
RV2478C		unknown	1.9	Up
RV2479C		insertion seqs and phages	2.98	Up
RV2481C		unknown	1.97	Up
RV2485C	lipQ	intermediary metabolism and respiration	2.26	Up
RV2486	echA14	lipid metabolism	1.98	Up
RV2488C		regulatory proteins	2.97	Up
RV2489C		unknown	3.59	Up
RV2490C	PE_PGRS	PE/PPE	18.38	Down
RV2491		unknown	3.67	Up
RV2492		unknown	2.42	Up
RV2495C	pdhC	intermediary metabolism and respiration	1.79	Up
RV2499C		intermediary metabolism and respiration	1.71	Up
RV2510C		conserved hypotheticals	2	Up
RV2513		unknown	2.9	Up
RV2518C	lppS	cell wall and cell processes	2.25	Up
RV2523C	acpS	lipid metabolism	2.25	Up
RV2524C	fas	lipid metabolism	4.04	Down
RV2529		unknown	2.23	Up
RV2533C	nusB	information pathways	1.71	Up
RV2540C	aroF	intermediary metabolism and respiration	2.11	Up
RV2541		unknown	2.45	Up
RV2543	lppA	cell wall and cell processes	3.06	Up
RV2544	lppB	cell wall and cell processes	2.89	Up
RV2545		conserved hypotheticals	3.06	Up

RV2548		conserved hypotheticals	2.92	Up
RV2553C		unknown	2.39	Down
RV2559C		conserved hypotheticals	2.7	Up
RV2560		cell wall and cell processes	2.69	Up
RV2561		unknown	2.25	Up
RV2562		unknown	2.66	Up
RV2566		unknown	2.72	Up
RV2567		conserved hypotheticals	2.63	Up
RV2568C		conserved hypotheticals	2.98	Up
RV2569C		conserved hypotheticals	2.47	Up
RV2570		unknown	2.54	Up
RV2571C		conserved hypotheticals	2.12	Up
RV2575		conserved hypotheticals	2.38	Up
RV2576C		unknown	3.78	Down
RV2577		unknown	2.46	Up
RV2579	linB	intermediary metabolism and respiration	2.95	Up
RV2584C	apt	intermediary metabolism and respiration	2.92	Down
RV2591	PE_PGRS	PE/PPE	20.16	Down
RV2593C	ruvA	information pathways	2.03	Up
RV2594C	ruvC	information pathways	4.72	Down
RV2603C		conserved hypotheticals	1.76	Up
RV2617C		unknown	1.8	Up
RV2622		intermediary metabolism and respiration	1.91	Up
RV2623		conserved hypotheticals	7.02	Down
RV2625C		conserved hypotheticals	9.43	Down
RV2626C		conserved hypotheticals	6.69	Down
RV2628		unknown	3	Down
RV2629		unknown	2.57	Down
RV2630		unknown	2.29	Down
RV2636		unknown	3.85	Up
RV2637	dedA	conserved hypotheticals	2.42	Up
RV2638		conserved hypotheticals	2.84	Up
RV2640C		regulatory proteins	1.58	Up
RV2646		insertion seqs and phages	2.23	Up
RV2653C		insertion seqs and phages	2.8	Up
RV2654C		insertion seqs and phages	2.66	Up
RV2655C		insertion seqs and phages	3.42	Up
RV2656C		insertion seqs and phages	2.09	Up
RV2658C		insertion seqs and phages	3.86	Up
RV2659C		insertion seqs and phages	1.93	Up
RV2660C		unknown	2.76	Up
RV2661C		unknown	2.57	Up
RV2662		unknown	2.7	Up
RV2667	clpX'	intermediary metabolism and respiration	2.45	Up
RV2669		regulatory proteins	2.05	Up
RV2670C		conserved hypotheticals	3.33	Up
RV2671	ribD	intermediary metabolism and respiration	1.78	Up
RV2676C		conserved hypotheticals	1.96	Up
RV2681		conserved hypotheticals	2.13	Up

RV2684	arsA	cell wall and cell processes	2.69	Up
RV2685	arsB	cell wall and cell processes	1.97	Up
RV2686C		cell wall and cell processes	2.62	Up
RV2687C		unknown	2.49	Up
RV2710	sigB	information pathways	7.07	Down
RV2712C		unknown	2.23	Up
RV2716		conserved hypotheticals	2.18	Down
RV2721C		conserved hypotheticals	3.85	Down
RV2728C		conserved hypotheticals	1.81	Up
RV2730		unknown	3.49	Up
RV2732C		conserved hypotheticals	1.54	Up
RV2743C		conserved hypotheticals	4.99	Down
RV2745C		regulatory proteins	6.72	Down
RV2750		intermediary metabolism and respiration	2.44	Up
RV2756C	hsdM	information pathways	2.71	Up
RV2758C		conserved hypotheticals	2.01	Up
RV2760C		conserved hypotheticals	3.55	Up
RV2761C		unknown	2.15	Down
RV2774C		unknown	2.9	Up
RV2780	ald	intermediary metabolism and respiration	2.76	Down
RV2797C		conserved hypotheticals	2.14	Up
RV2798C		conserved hypotheticals	1.79	Up
RV2799		unknown	2.45	Up
RV2800		conserved hypotheticals	1.9	Up
RV2803C		unknown	2.9	Up
RV2804C		unknown	2.26	Up
RV2805		conserved hypotheticals	2.61	Up
RV2807		conserved hypotheticals	2	Up
RV2811		conserved hypotheticals	4.02	Down
RV2823C		conserved hypotheticals	2.01	Up
RV2825C		conserved hypotheticals	2.11	Up
RV2831	echA16	lipid metabolism	1.84	Up
RV2834C	ugpE	cell wall and cell processes	2.16	Up
RV2836C	dinF	information pathways	2.21	Down
RV2838C	rbfA	information pathways	2.83	Down
RV2840C		conserved hypotheticals	14.39	Down
RV2845C	proS	information pathways	1.76	Up
RV2846C	efpA	cell wall and cell processes	4.41	Down
RV2851C		conserved hypotheticals	2.08	Up
RV2853	PE_PGRS	PE/PPE	2.57	Up
RV2854		unknown	2.56	Up
RV2856	nicT	cell wall and cell processes	2.33	Up
RV2862C		conserved hypotheticals	4.52	Down
RV2864C		cell wall and cell processes	1.79	Up
RV2866		conserved hypotheticals	2.79	Up
RV2871		conserved hypotheticals	1.73	Up
RV2873	mpt83	cell wall and cell processes	2.34	Up
RV2874		cell wall and cell processes	2.4	Up
RV2875	mpt70	cell wall and cell processes	2.43	Up

RV2877C		cell wall and cell processes	2.01	Up
RV2886C		insertion seqs and phages	2.21	Down
RV2891		unknown	2.43	Up
RV2892C	PPE	PE/PPE	2.01	Up
RV2893		intermediary metabolism and respiration	2.94	Up
RV2895C	viuB	intermediary metabolism and respiration	2.73	Up
RV2896C		conserved hypotheticals	2.83	Up
RV2897C		conserved hypotheticals	3.03	Up
RV2898C		conserved hypotheticals	2.67	Up
RV2899C	fdhD	intermediary metabolism and respiration	2.37	Up
RV2901C		unknown	2.07	Down
RV2902C	rnhB	intermediary metabolism and respiration	2.15	Down
RV2907C	rimM	information pathways	2.05	Up
RV2917		conserved hypotheticals	3.36	Up
RV2920C	amt	cell wall and cell processes	1.96	Up
RV2922C	smc	cell wall and cell processes	1.83	Up
RV2923C		conserved hypotheticals	2.44	Up
RV2924C	fpg	information pathways	3.21	Up
RV2927C		conserved hypotheticals	1.57	Down
RV2928	tesA	lipid metabolism	2.65	Up
RV2930	fadD26	lipid metabolism	2.43	Down
RV2932	ppsB	lipid metabolism	1.73	Up
RV2934	ppsD	lipid metabolism	2.19	Up
RV2935	ppsE	lipid metabolism	15.35	Down
RV2938	drrC	cell wall and cell processes	2.13	Up
RV2940C	mas	lipid metabolism	2.57	Down
RV2943		insertion seqs and phages	2.04	Up
RV2960C		unknown	1.92	Up
RV2962C		intermediary metabolism and respiration	2.9	Up
RV2963		cell wall and cell processes	2.47	Up
RV2965C	kdtB	cell wall and cell processes	2.41	Up
RV2972C		unknown	3.05	Up
RV2973C	recG	information pathways	2.89	Up
RV2975C		conserved hypotheticals	3.32	Up
RV2980		unknown	2.08	Up
RV2983		conserved hypotheticals	2.4	Up
RV2986C	hupB	information pathways	3.4	Down
RV2988C	leuC	intermediary metabolism and respiration	6.79	Down
RV2989		regulatory proteins	2.11	Down
RV2990C		unknown	1.91	Down
RV2991		conserved hypotheticals	2.35	Up
RV2992C	gltS	information pathways	2.43	Up
RV2995C	leuB	intermediary metabolism and respiration	3.39	Up
RV2997		intermediary metabolism and respiration	2.57	Up
RV2998		unknown	2.51	Up
RV2999	lppY	cell wall and cell processes	2.96	Up
RV3000		conserved hypotheticals	3.62	Up
RV3007C		unknown	1.72	Up
RV3011C	gatA	information pathways	3.07	Up

RV3012C	gatC	information pathways	2.23	Up
RV3015C		conserved hypotheticals	2.81	Up
RV3016	lpqA	cell wall and cell processes	2.94	Up
RV3018C	PPE	PE/PPE	3.25	Up
RV3019C		cell wall and cell processes	1.59	Down
RV3020C	PE	PE/PPE	2.05	Down
RV3021C	PPE	PE/PPE	3.26	Up
RV3027C		unknown	2.45	Up
RV3031		conserved hypotheticals	2.09	Up
RV3032		conserved hypotheticals	2.2	Up
RV3033		unknown	1.79	Up
RV3034C		conserved hypotheticals	1.8	Up
RV3039C	echA17	lipid metabolism	2.57	Up
RV3040C		unknown	2.25	Up
RV3048C	nrdG	intermediary metabolism and respiration	3.93	Up
RV3049C		intermediary metabolism and respiration	2.77	Down
RV3050C		regulatory proteins	2.25	Down
RV3053C	nrdH	intermediary metabolism and respiration	6.71	Down
RV3055		regulatory proteins	2.64	Up
RV3057C		intermediary metabolism and respiration	1.98	Up
RV3059		intermediary metabolism and respiration	2.5	Up
RV3062	ligB	information pathways	2.5	Up
RV3064C		conserved hypotheticals	2.75	Up
RV3066		regulatory proteins	2.25	Up
RV3067		conserved hypotheticals	2.26	Up
RV3068C	pgmA	intermediary metabolism and respiration	2.1	Up
RV3070		cell wall and cell processes	2.99	Up
RV3083		intermediary metabolism and respiration	5.93	Down
RV3084	lipR	intermediary metabolism and respiration	2.62	Down
RV3090		unknown	2.05	Up
RV3094C		conserved hypotheticals	1.54	Down
RV3096		unknown	1.89	Up
RV3101C	ftsX	cell wall and cell processes	1.61	Down
RV3108		unknown	2.09	Up
RV3109	moaA	intermediary metabolism and respiration	2.56	Up
RV3110	moaB	intermediary metabolism and respiration	3.25	Up
RV3112	moaD	intermediary metabolism and respiration	3.24	Up
RV3113		unknown	4.6	Up
RV3114		unknown	3.48	Up
RV3119	moaE	intermediary metabolism and respiration	2.89	Up
RV3124		regulatory proteins	2.72	Up
RV3127		conserved hypotheticals	4.78	Down
RV3129		conserved hypotheticals	4.34	Up
RV3130C		conserved hypotheticals	6.46	Down
RV3131		conserved hypotheticals	10.8	Down
RV3134C		conserved hypotheticals	2.2	Down
RV3138	pflA	intermediary metabolism and respiration	1.91	Up
RV3142C		unknown	1.95	Down
RV3145	nuoA	intermediary metabolism and respiration	2.1	Down

RV3149	nuoE	intermediary metabolism and respiration	2.97	Down
RV3155	nuoK	intermediary metabolism and respiration	1.74	Down
RV3158	nuoN	intermediary metabolism and respiration	1.66	Up
RV3160C		regulatory proteins	1.86	Down
RV3161C		intermediary metabolism and respiration	2.52	Down
RV3162C		cell wall and cell processes	1.92	Down
RV3165C		unknown	3.03	Up
RV3181C		conserved hypotheticals	2.47	Up
RV3182		conserved hypotheticals	1.67	Up
RV3187		insertion seqs and phages	3.06	Up
RV3189		conserved hypotheticals	2.1	Up
RV3190C		unknown	1.8	Up
RV3191C		insertion seqs and phages	2.75	Up
RV3194C		conserved hypotheticals	1.74	Up
RV3197		cell wall and cell processes	4.71	Down
RV3204		intermediary metabolism and respiration	1.8	Up
RV3210C		unknown	2.22	Up
RV3211	rhlE	information pathways	1.8	Down
RV3212		unknown	2.15	Down
RV3214	entD	intermediary metabolism and respiration	2.27	Up
RV3215	entC	intermediary metabolism and respiration	2.31	Up
RV3216		intermediary metabolism and respiration	1.5	Up
RV3218		conserved hypotheticals	2.59	Up
RV3220C		regulatory proteins	2.93	Down
RV3223C	sigH	information pathways	4.27	Up
RV3224		intermediary metabolism and respiration	2.51	Down
RV3228		conserved hypotheticals	1.59	Up
RV3229C	desA3	lipid metabolism	2.81	Up
RV3231C		unknown	2.59	Up
RV3232C		conserved hypotheticals	1.64	Up
RV3233C		conserved hypotheticals	2	Up
RV3235		unknown	2.2	Up
RV3239C		cell wall and cell processes	2.16	Up
RV3249C		regulatory proteins	7.7	Down
RV3250C	rubB	intermediary metabolism and respiration	12.22	Down
RV3251C	rubA	intermediary metabolism and respiration	6.22	Down
RV3252C		intermediary metabolism and respiration	14.93	Down
RV3253C		cell wall and cell processes	1.75	Up
RV3255C	manA	intermediary metabolism and respiration	2.14	Up
RV3259		unknown	2.71	Up
RV3265C	wbbL	intermediary metabolism and respiration	1.66	Up
RV3266C	rmlD	intermediary metabolism and respiration	2.87	Up
RV3275C	purE	intermediary metabolism and respiration	2.35	Up
RV3279C	birA	intermediary metabolism and respiration	3.53	Up
RV3281		conserved hypotheticals	2.3	Down
RV3283	sseA	intermediary metabolism and respiration	2.25	Down
RV3287C	rsbW	information pathways	1.65	Down
RV3290C	lat	intermediary metabolism and respiration	4.2	Down
RV3294		unknown	1.81	Up

RV3295		regulatory proteins	2.13	Down
RV3299C	atsB	intermediary metabolism and respiration	1.92	Up
RV3302C	glpD2	intermediary metabolism and respiration	2.32	Up
RV3309C	upp	intermediary metabolism and respiration	2.91	Up
RV3311		conserved hypotheticals	2.71	Up
RV3312C		conserved hypotheticals	3.19	Up
RV3314C	deoA	intermediary metabolism and respiration	2.95	Up
RV3324C	moaC3	intermediary metabolism and respiration	3.36	Up
RV3326		insertion seqs and phages	3.56	Up
RV3327		insertion seqs and phages	2.8	Up
RV3329		intermediary metabolism and respiration	2.93	Up
RV3330		cell wall and cell processes	2.11	Up
RV3331	sugI	cell wall and cell processes	1.57	Up
RV3333C		unknown	3.55	Up
RV3336C	trpS	information pathways	2.92	Up
RV3339C	icd1	intermediary metabolism and respiration	1.79	Up
RV3343C	PPE	PE/PPE	3.46	Up
RV3347C	PPE	PE/PPE	1.69	Up
RV3350C	PPE	PE/PPE	3.74	Up
RV3351C		conserved hypotheticals	3.66	Up
RV3352C		intermediary metabolism and respiration	3.55	Up
RV3353C		conserved hypotheticals	3.42	Up
RV3354		conserved hypotheticals	2.77	Up
RV3355C		conserved hypotheticals	2.38	Up
RV3357		conserved hypotheticals	3.54	Up
RV3358		unknown	3.34	Up
RV3359		intermediary metabolism and respiration	2.86	Up
RV3360		cell wall and cell processes	2.65	Up
RV3362C		unknown	2.11	Up
RV3363C		unknown	1.95	Up
RV3366	spoU	information pathways	2.87	Up
RV3370C	dnaE2	information pathways	2.69	Up
RV3372	otsB2	virulence, detoxification, adaptation	1.94	Up
RV3374	echA18'	lipid metabolism	3.59	Up
RV3377C		intermediary metabolism and respiration	1.76	Up
RV3378C		unknown	3	Up
RV3379C		intermediary metabolism and respiration	2.22	Up
RV3380C		insertion seqs and phages	3.35	Up
RV3383C	idsB	intermediary metabolism and respiration	2.23	Up
RV3384C		conserved hypotheticals	3	Up
RV3387		insertion seqs and phages	2.48	Up
RV3388	PE_PGRS	PE/PPE	5.27	Down
RV3391	acrA1	lipid metabolism	1.96	Up
RV3392C	cmaA1	lipid metabolism	2.02	Up
RV3395C		unknown	2.46	Up
RV3398C	idsA	intermediary metabolism and respiration	2.84	Up
RV3399		conserved hypotheticals	3.33	Up
RV3400		intermediary metabolism and respiration	2.33	Up
RV3402C		cell wall and cell processes	3.48	Down

RV3404C		unknown	1.8	Up
RV3406		intermediary metabolism and respiration	3.3	Up
RV3407		conserved hypotheticals	8.9	Down
RV3408		conserved hypotheticals	7.03	Down
RV3411C	guaB2	intermediary metabolism and respiration	4.73	Down
RV3412		conserved hypotheticals	2.46	Down
RV3415C		conserved hypotheticals	2.28	Up
RV3417C	groEL1	virulence, detoxification, adaptation	7.18	Down
RV3421C		conserved hypotheticals	3.49	Down
RV3430C		insertion seqs and phages	2.53	Up
RV3431C		insertion seqs and phages	2.87	Up
RV3436C	glmS	intermediary metabolism and respiration	1.6	Up
RV3444C		conserved hypotheticals	2.77	Up
RV3446C		unknown	3.27	Up
RV3447C		cell wall and cell processes	2.77	Up
RV3448		cell wall and cell processes	3.78	Up
RV3449		cell wall and cell processes	1.76	Up
RV3452		cell wall and cell processes	2.53	Up
RV3453		unknown	2.58	Up
RV3454		cell wall and cell processes	2.96	Up
RV3458C	rpsD	information pathways	3.28	Down
RV3459C	rpsK	information pathways	6.91	Down
RV3468C	rmlB3	intermediary metabolism and respiration	3.1	Up
RV3469C	mhpE	intermediary metabolism and respiration	2.47	Up
RV3470C	ilvB2	intermediary metabolism and respiration	2.62	Up
RV3471C		conserved hypotheticals	3.73	Up
RV3472		lipid metabolism	1.64	Up
RV3473C	bpoA	virulence, detoxification, adaptation	3.58	Up
RV3475		insertion seqs and phages	3.77	Up
RV3476C	kgtP	cell wall and cell processes	3.36	Up
RV3477	PE	PE/PPE	6.45	Down
RV3478	PPE	PE/PPE	12.63	Down
RV3479		unknown	1.65	Up
RV3480C		conserved hypotheticals	2.53	Up
RV3487C	lipF	intermediary metabolism and respiration	19.26	Down
RV3489		unknown	4.32	Down
RV3493C		unknown	2.04	Up
RV3494C		virulence, detoxification, adaptation	1.93	Up
RV3496C		virulence, detoxification, adaptation	1.81	Up
RV3498C		virulence, detoxification, adaptation	2.03	Up
RV3500C		virulence, detoxification, adaptation	2.33	Up
RV3501C		virulence, detoxification, adaptation	2.12	Up
RV3502C		intermediary metabolism and respiration	2.89	Up
RV3508	PE_PGRS	PE/PPE	31.42	Down
RV3512	PE_PGRS	PE/PPE	17.08	Down
RV3517		conserved hypotheticals	2.24	Up
RV3528C		unknown	1.84	Up
RV3529C		conserved hypotheticals	1.79	Up
RV3534C		intermediary metabolism and respiration	2.6	Up

RV3537		intermediary metabolism and respiration	2.48	Up
RV3538	ufaA2	lipid metabolism	2.27	Up
RV3541C		unknown	2.75	Up
RV3542C		unknown	2.23	Up
RV3543C	fadE29	lipid metabolism	2.63	Up
RV3544C	fadE28	lipid metabolism	2.4	Up
RV3545C		intermediary metabolism and respiration	2.03	Up
RV3546	fadA5	lipid metabolism	2.9	Up
RV3550	echA20	lipid metabolism	1.65	Up
RV3552		unknown	1.91	Up
RV3554	fdxB	intermediary metabolism and respiration	2.36	Down
RV3556C	fadA6	lipid metabolism	2.77	Down
RV3559C		intermediary metabolism and respiration	2.32	Up
RV3565	aspB	intermediary metabolism and respiration	2.6	Up
RV3567C		intermediary metabolism and respiration	2.52	Up
RV3569C		intermediary metabolism and respiration	1.73	Up
RV3571		intermediary metabolism and respiration	2.51	Up
RV3572		unknown	2.22	Up
RV3573C	fadE34	lipid metabolism	2.13	Up
RV3574		regulatory proteins	2.91	Up
RV3575C		regulatory proteins	2.45	Up
RV3582C		conserved hypotheticals	4.07	Down
RV3583C		regulatory proteins	7.59	Down
RV3589	mutY	information pathways	2.47	Up
RV3590C	PE_PGRS	PE/PPE	2	Down
RV3591C		conserved hypotheticals	3.07	Up
RV3593	lpqF	cell wall and cell processes	1.75	Up
RV3594		unknown	2.16	Up
RV3595C	PE_PGRS	PE/PPE	4.69	Down
RV3597C	lsr2	conserved hypotheticals	2.12	Up
RV3599C		unknown	2.17	Up
RV3611		unknown	2.41	Up
RV3613C		unknown	4.29	Down
RV3614C		conserved hypotheticals	11.28	Down
RV3615C		conserved hypotheticals	15.1	Down
RV3616C		conserved hypotheticals	9.52	Down
RV3619C		conserved hypotheticals	11.17	Down
RV3620C		conserved hypotheticals	28.48	Down
RV3621C	PPE	PE/PPE	1.78	Up
RV3629C		cell wall and cell processes	2.38	Down
RV3636		insertion seqs and phages	2.38	Up
RV3639C		conserved hypotheticals	2.09	Up
RV3641C	fic	cell wall and cell processes	1.58	Up
RV3643		unknown	2.98	Up
RV3644C		information pathways	2.36	Up
RV3645		cell wall and cell processes	1.84	Up
RV3648C	cspA	virulence, detoxification, adaptation	6.41	Down
RV3650	PE	PE/PPE	2.19	Up
RV3652	PE_PGRS	PE/PPE	2.25	Up

RV3653	PE_PGRS	PE/PPE	10.06	Down
RV3654C		unknown	2.35	Up
RV3658C		cell wall and cell processes	2.08	Up
RV3659C	trbB	cell wall and cell processes	3	Up
RV3664C	dppC	cell wall and cell processes	3.04	Up
RV3670	ephE	virulence, detoxification, adaptation	3.58	Up
RV3676		regulatory proteins	2.37	Up
RV3682	ponA'	cell wall and cell processes	1.7	Down
RV3685C		intermediary metabolism and respiration	2.06	Up
RV3691		unknown	2.32	Up
RV3693		conserved hypotheticals	2.29	Up
RV3695		conserved hypotheticals	2.79	Up
RV3696C	glpK	intermediary metabolism and respiration	3.05	Up
RV3700C		intermediary metabolism and respiration	2.77	Up
RV3705C		unknown	1.63	Up
RV3706C		unknown	3.09	Down
RV3709C	ask	intermediary metabolism and respiration	1.67	Up
RV3711C	dnaQ	information pathways	2.17	Up
RV3712		conserved hypotheticals	2.67	Up
RV3715C	recR	information pathways	2.51	Up
RV3720		lipid metabolism	2.16	Up
RV3723		unknown	1.61	Up
RV3743C		cell wall and cell processes	2.99	Up
RV3745C		conserved hypotheticals	2.13	Up
RV3746C	PE	PE/PPE	2.35	Up
RV3749C		unknown	3.09	Down
RV3751		insertion seqs and phages	2.31	Up
RV3761C	fadE36	lipid metabolism	2.04	Up
RV3764C		regulatory proteins	2.58	Up
RV3768		unknown	3.06	Up
RV3771C		conserved hypotheticals	1.67	Up
RV3772	hisC2	intermediary metabolism and respiration	1.9	Up
RV3773C		conserved hypotheticals	2.03	Up
RV3775	lipE	intermediary metabolism and respiration	1.82	Up
RV3780		unknown	1.83	Up
RV3781		cell wall and cell processes	1.76	Up
RV3784	epiB	intermediary metabolism and respiration	2.71	Up
RV3785		unknown	2.35	Up
RV3786C		unknown	3.52	Up
RV3788		unknown	2	Up
RV3790		intermediary metabolism and respiration	2.75	Up
RV3791		intermediary metabolism and respiration	2.58	Up
RV3792		cell wall and cell processes	3.27	Up
RV3796	atsH	intermediary metabolism and respiration	2.5	Up
RV3797	fadE35	lipid metabolism	2.37	Up
RV3800C	pks13	lipid metabolism	5.07	Down
RV3801C	fadD32	lipid metabolism	7.05	Down
RV3803C	fbpC1	lipid metabolism	1.67	Down
RV3804C	fbpA	lipid metabolism	17.36	Down

RV3806C		cell wall and cell processes	2.1	Up
RV3809C	glf	cell wall and cell processes	1.83	Up
RV3813C		conserved hypotheticals	1.98	Up
RV3818		unknown	2.38	Down
RV3819		unknown	1.63	Up
RV3822		conserved hypotheticals	4.73	Down
RV3825C	pks2	lipid metabolism	1.7	Down
RV3828C		insertion seqs and phages	2.62	Down
RV3830C		regulatory proteins	2.58	Up
RV3832C		unknown	2.2	Up
RV3834C	serS	information pathways	3.16	Up
RV3835		unknown	1.78	Down
RV3841	bfrB	intermediary metabolism and respiration	32.32	Down
RV3849		unknown	5.44	Down
RV3853	menG	intermediary metabolism and respiration	2.14	Up
RV3860		conserved hypotheticals	2.03	Up
RV3861		unknown	3.07	Up
RV3862C		unknown	2.06	Up
RV3863		unknown	2.29	Up
RV3865		conserved hypotheticals	5.93	Down
RV3867		conserved hypotheticals	4.94	Down
RV3869		conserved hypotheticals	7.58	Down
RV3870		conserved hypotheticals	4.2	Down
RV3871		conserved hypotheticals	5.29	Down
RV3872	PE	PE/PPE	4.81	Down
RV3873	PPE	PE/PPE	5.28	Down
RV3874		conserved hypotheticals	20.56	Down
RV3875	esat6	cell wall and cell processes	23.87	Down
RV3876		conserved hypotheticals	2.82	Down
RV3879C		unknown	1.67	Down
RV3880C		conserved hypotheticals	1.51	Down
RV3884C		conserved hypotheticals	1.69	Up
RV3894C		cell wall and cell processes	2.54	Up
RV3901C		cell wall and cell processes	3.04	Up
RV3902C		unknown	3.06	Up
RV3903C		unknown	2.91	Up
RV3904C		unknown	1.91	Up
RV3907C	pcnA	information pathways	2.42	Up
RV3908		conserved hypotheticals	2.24	Up
RV3909		conserved hypotheticals	2.35	Up
RV3911	sigM	information pathways	2.64	Up
RV3912		unknown	1.9	Up
RV3914	trxC	intermediary metabolism and respiration	2.16	Down
RV3919C	gid	cell wall and cell processes	3.4	Down
RV3921C		cell wall and cell processes	9.06	Down
RV3923C	rnpA	intermediary metabolism and respiration	4.35	Down
