# Supplementary material for: MazF6 toxin of Mycobacterium tuberculosis demonstrates antitoxin specificity and is coupled to regulation of cell growth by a Soj-like protein
Source: BMC Microbiol. 2013 Oct 31;13:240. doi: 10.1186/1471-2180-13-240 (PMC3834876; doi:10.1186/1471-2180-13-240)
Supplement: Additional file 1 — Primer sequences used for plasmid construction. [file 1471-2180-13-240-S1.pdf]

Additional file 2: Primer sequences used for plasmid construction

Gene	Rv #		Primer Sequence
soj_{Mtb}	*rv1708*	Forward	5’ - CTGGTACATATGTTGCCTGCGGGTCTCCC
		Reverse	5’ - AAGCTCAAGCTTCATGCCAAATCGGTCGATC
mazE6	*rv1991a*	Forward	5’ - GGTCGCGGATCCATGAAGACAGCTATTTCTCTG
		Reverse	5’ - GTGGTGCTCGAGCCACTCATCGTCCATGGT
mazF6	*rv1991c*	Forward	5’ - CCCTTTGCATGCTCATGGTGATTAGTCGTG
		Reverse	5’ - GGGTTTGCGGCCGCAAGGTCCAGTACGCG
mazE1	*rv0456b*	Forward	5’ - GGTCGCGGATCCATGACTACCTATTACTACG
		Reverse	5’ - GTGGTGAAGCTTCCGCGCAGCATCTACG
mazE2	*rv0660c*	Forward	5’ - GGTCGCGGATCCATGCTCAGCTTCCGCGC
		Reverse	5’ - GTGGTGAAGCTTCCGCGCCGCATCGGC
mazE3	*rv1103c*	Forward	5’ - GGTCGCGGATCCATGTACCTACCCTGGGG
		Reverse	5’ - GTGGTGCTCGAGGTCGATGTCGAGGGCG
mazE4	*rv1494*	Forward	5’ - GGTCGCGGATCCATGCCGTTTCTGGTTGCATT
		Reverse	5’ - GTGGTGCTCGAGCTGTGCCGAGCGCTGC
mazE5	*rv1943c*	Forward	5’ - GGTCGCGAATTCATGAAGACGGCCCGGTTG
		Reverse	5’ - GTGGTGCTCGAGCGAGTCTCCAGCCGCC
mazE7	*rv2063*	Forward	5’ - GGTCGCGGATCCATGTCTACATCCACGACG
		Reverse	5’ - GTGGTGAAGCTTGCCAAGGCCGTCGCC
mazE9	*rv2801a*	Forward	5’ - GGTCGCGGATCCATGAAGTTGAGCGTGAGC
		Reverse	5’ - GTGGTGAAGCTTCCGCGGCGCATCACC
