# Supplementary material for: MazF6 toxin of Mycobacterium tuberculosis demonstrates antitoxin specificity and is coupled to regulation of cell growth by a Soj-like protein
Source: BMC Microbiol. 2013 Oct 31;13:240. doi: 10.1186/1471-2180-13-240 (PMC3834876; doi:10.1186/1471-2180-13-240)
Supplement: Additional file 3 — Primer sequences used for end-point PCR. [file 1471-2180-13-240-S3.pdf]

Additional file 3: Primer sequences used for end-point PCR

Gene	Rv #		Primer Sequence
mazE6	*rv1991a*	Forward	5' – ATGAGTCGGTCCGAGTTCTTCACG
		Reverse	5' – TCCATGGTTTCTAGCACGCGGTAT
mazF6	*rv1991c*	Forward	5' – TCCAGTCAGATCCGTACAACGCAA
		Reverse	5' – AGGTCAGCTTTGTTGAGCGTGACA
sigA	*rv2703*	Forward	5' – TTCGCGCCTACCTCAAACAG
		Reverse	5' – GCTAGCTCGACCTCTTCCTCG
